# Vocal sequence diversity and length remain stable across ontogeny in a catarrhine monkey (*Cercocebus atys*)

**DOI:** 10.1038/s42003-025-07922-2

**Published:** 2025-03-20

**Authors:** Ryan Sigmundson, Cédric Girard-Buttoz, Auriane Le Floch, Tanit Souha Azaiez, Richard McElreath, Klaus Zuberbühler, Roman M. Wittig, Catherine Crockford

**Affiliations:** 1 The Ape Social Mind Lab, Institut des Sciences Cognitives Marc Jeannerod, CNRS, Bron, Lyon France; 2 Taï Chimpanzee Project, Centre Suisse de Recherches Scientifique en Côte d’Ivoire, Abidjan, Ivory Coast; 3 ENES Bioacoustics Research Laboratory, Centre de Recherche en Neurosciences de Lyon, CNRS, Inserm, University of Saint-Etienne, Saint-Etienne, France; 4 Department of Human Behaviour, Ecology and Culture, Max Planck Institute for Evolutionary Anthropology, Leipzig, Germany; 5 Institute of Biology, University of Neuchâtel, Neuchâtel, Switzerland; 6 Taï Monkey Project, Centre Suisse de Recherches Scientifique en Côte d’Ivoire, Abidjan, Ivory Coast; 7 School of Psychology & Neuroscience, University of St Andrews, St Andrews, Scotland

**Keywords:** Animal behaviour, Social anthropology

## Abstract

During childhood, human speech utterances increase steadily in complexity, length and diversity. In contrast, the vocal repertoire of non-human primates has long been considered fixed from birth. Recent studies showing the acquisition of vocal sequences during ontogeny in chimpanzees and marmosets challenge this view. Here we further explore the potential flexibility of non-human primate vocal production by comparing the vocal sequence repertoire across age groups in sooty mangabeys, a species with a rich sequence repertoire for a catarrhine monkey. We recorded 1844 utterances from 75 individuals from two wild groups in Taï National Park, Ivory Coast. We used custom-made Bayesian models specifically designed to estimate the individual repertoire size of vocal sequences while accounting for under-sampling of certain vocalisations in certain individuals. We hereby provide a tool to estimate vocal repertoire size applicable to other taxa. We found no relevant ontogenetic changes in vocal repertoire size and utterance length. Ontogenetic vocal sequence expansion is therefore not universal among primates that routinely use vocal sequences to communicate. Rather, this feature may have evolved independently in distantly-related taxa due to social features thought to promote vocal complexity, such as the complex social organisation of chimpanzees and the cooperative breeding systems of marmosets.

## Abstract (web summary)

An observational study on wild mangabeys using a Bayesian method to estimate vocal repertoire size finds that vocal sequence diversity and length remain stable throughout ontogeny, indicating a lack of vocal usage learning.

## Introduction

The human capacity to generate meaning is not so much grounded in the range of sounds the vocal tract is able to produce, but in its capacity for rapid and finely-controlled sound combinations^1–3^. This open-ended combinatorial ability enables speech, an unparalleled vocal engine that is part of what defines us as a species^3^. Yet, many animals also combine communication signals into sequences, suggesting that human speech may be an extreme endpoint of a more basic evolutionary continuum. In non-human primates, vocal sequence production has been documented, so far, in 31 species across the major clades, i.e., Prosimians, Platyrrhini, Cercopithecidae and Hominidae^4^. In at least some taxa vocal sequences are functionally relevant and convey specific information to the receivers^5–11^. Yet, most studies focus on a few vocal sequences per species and systematic examination of the sequence repertoire has only been carried out in a handful of species^12–18^. Within the cercopithecines (formally labelled as Old World monkeys), studies of sequence usage have predominantly focused on bigrams (two-unit vocal sequences) in alarm contexts^6,8–11,13,18^ (but see ref. ^19^), identifying combinatorial mechanisms such as affixation^6,8,9,11,18^. For evolutionary considerations this narrow perspective on vocal sequence usage is problematic, especially if the goal is to retrace the evolution of combinatorial capacities that, in one way or another, paved the way to speech.

Humans are not only unique in their open-ended sequence repertoire but also in their underlying semantic and syntactic capacities and how they develop. It takes at least seven years until children are able to use utterances containing adult-like speech, with increases in complexity continuing to develop well into adulthood^20^. This increase in complexity is often contrasted with primate vocal development, which is drastically more limited. Monkey vocal repertoire studies however have focused on the emergence of single calls, as these are predominant in most repertoires, with extremely few studies examining ontogenetic emergence of vocal sequences (but see^21^). Single calls typically emerge early and remain stable over the entire lifespan^22–24^. Other often cited examples are the highly invasive laboratory studies with infant squirrel monkeys (*Saimiri sciureus*) that were either deafened or raised by muted parents and nevertheless produced the species-typical vocalizations^23,25^. Such studies have led to the widely accepted conclusion that non-human primates have very little capacity for vocal production learning (the production of new signals in response to experience^26^) and no real ability to create new structures beyond small acoustic modifications of the basic call types^27–30^, which has been interpreted as vocal repertoire innateness and genetic determination^29,31^.

While the ability for vocal production learning of non-human primates contrasts drastically with that of humans^27,28^, other primates are capable of another form of vocal learning, vocal *contextual* or *usage learning* (e.g., see refs. ^32–35^, reviewed in^36–38^), which refers to the ability to use existing signals flexibly in different situations^26,39^. Young primates in several taxa were shown to be able to learn to use a specific call type in a certain context (e.g., see refs. ^34,40,41^, reviewed in^36–38^). Vocal usage learning can also refer to the ability to learn to recombine existing calls into sequences since no acoustic modification of the existing sound set is required^26,42,43^. There is, however, little knowledge about the specific mechanisms through which animals learn to use calls in sequences, and on how widespread this form of learning is in the animal kingdom in general and in primates in particular^42,43^. Usage learning of vocal sequences has been experimentally demonstrated in songbirds, which can learn to modify the temporal arrangement of signals^44,45^. In the primate order, vocal usage learning of vocal sequences has not yet been formally demonstrated, but recent studies strongly suggest that this form of learning could be present in at least two species, common marmosets (*Callithrix jacchus*)^21^ and chimpanzees (*Pan troglodytes*)^46^. These two species produce a large diversity of vocal sequences and are the only species where embedding of short sequences (two-unit sequence, bigrams) in larger sequences has been formally demonstrated^4,47^.

Young chimpanzees expand both the diversity and the length of their vocal sequences during ontogeny, with incremental changes from infancy to adolescence and adulthood^48^. The same set of single call types are found across a wide range of chimpanzee populations^49^ and all single calls are produced early in ontogeny, around 4 years of age^48^. Yet, vocal sequences continue to be acquired much later in ontogeny, at least until 8–9 years of age when chimpanzees begin to gain social independence from their mother. The trajectory of this protracted development of vocal sequences in chimpanzees thus presents some parallel to human speech development^20^. A similar case can be made for a platyrrhine cooperative breeding monkey: the common marmosets. All major call types emerge in the species within the first month of life but their use of vocal sequences retains a high degree of plasticity until adulthood^21^. It is not possible as of yet to fully exclude maturation as a process driving these observed ontogenetic trajectories. However, at least for chimpanzees, this possibility appears unlikely and vocal usage learning seems the most parsimonious explanation for these patterns. In support of this point, two populations of chimpanzees have been observed to produce bigrams in which a long-distance contact call and a submissive call are reordered in the sequence, despite similar contextual usage and thus potential function of the bigram^46^.

When solely considering the production of single calls, chimpanzee and marmoset vocal repertoires appear, as for other primates, mostly innate and inflexible. Only the investigation of vocal sequences gives rise to the possibility of vocal usage learning of vocal sequences in these species. It may be that the capacity for vocal learning through vocal sequence development evolved independently in (at least) these two species, constituting a case of convergent evolution, but is otherwise absent in the primate order. Certain complex social features that characterise chimpanzees and marmosets can drive vocal complexity^50–52^. Marmosets are cooperative breeders, which is hypothesised to be linked to volubility in communicative signals, particularly in the case of vocal signals^53,54^. Though chimpanzees are not cooperative breeders, they do engage in dyadic and group-level cooperative endeavors in multiple contexts with multiple goals^55,56^, indicating a high degree of social complexity. Another possibility is that ontogenetic vocal sequence expansion is present across the primate order and the potential capacity for vocal usage learning has been thus far overlooked due to overreliance on studies of single call production. However, further exploration of vocal sequence development in primates is needed to determine which of these explanations is correct. To further investigate the possibility of vocal usage learning through vocal sequence development in primates, catarrhine monkeys, the great apes’ closest relatives, are of interest, especially species living in similar socio-ecological conditions to chimpanzees. To address this, we studied the vocal sequence production of a terrestrial, forest-dwelling monkey, the sooty mangabey (*Cercocebus atys*), which lives sympatrically with chimpanzees. Sooty mangabeys form large multi-male, multi-female groups, similar to chimpanzees, and forage in large forested home ranges on a diverse mainly frugivorous diet^57^. These socio-ecological factors are thought to have a strong impact on a species’ communication behaviour and should favour increased vocal complexity^58,59^, suggesting that sooty mangabeys are an ideal comparison species to chimpanzees^48^. The species’ vocal repertoire has been described by Range and Fischer^60^, who documented 19 calls, although not all defined in terms of structure. Sooty mangabeys also make use of multimodal signal combinations, especially during social uncertainty^61^, something that has also been reported from closely related red-capped mangabeys (*C. torquatus*^19,62^), demonstrating the presence of rich sequence repertoires in mangabeys.

We aim to investigate whether the assumption of primate vocal repertoire innateness holds up when examined through the lens of vocal sequence usage across ontogeny. To this end, we use two measures of vocal sequence complexity, vocal repertoire size and maximum sequence length. Our prediction is that if vocal sequence complexity increases across the lifespan, then (a) vocal repertoire size (i.e., the number of different utterance types, including both vocalizations produced singly and in vocal sequences) should increase with age, and (b) sequence length should increase with age. Documenting such an increase would challenge the current understanding of primate repertoire stability.

To test these hypotheses, we collected vocal recordings during 784.6 h of focal observation on 75 wild mangabeys aged 1 month to 16 years from two social groups in the Taï National Park, Ivory Coast. The mangabeys were fully habituated to human observers and we used a systemic whole repertoire approach where vocalizations were continuously recorded during focal animal sampling^63–65^.

## Results

In our sample of mangabey recordings, we identified nine call types (Figures S1–S9) that corresponded to the broad vocalization categories in the sooty mangabey vocal repertoire laid out by Range and Fischer^60^, with the addition of the whimper (Figure S9). Of these, six were categorised as basic call types: grunt (GR), twitter (TW), scream (SM), growl (GO), whimper (WH), and hoo (HO). In addition, we considered three complex calls that contained multiple distinct elements that reliably occurred together but were never emitted independently and were thus treated as a single call. These complex calls included the long call (LC; sometimes referred to as the whoop gobble), the shrill call (SC; also referred to as alarm call), and the vibrato (VI; also referred to as copulation call).

Breaking down call usage by age class, we found that infants produced 6 different call types (either singly or within vocal sequences), while juveniles, subadults and adults produced 6, 6, and 7 different call types, respectively. Certain call types were specific to certain sexes or age classes while others were present across the lifespan and in both males and females (Figure S10). Grunt, twitter, scream, growl, and shrill call were used by all age classes (though adult males did not produce the twitter). Whimper was only produced by infants and juveniles, while the vibrato was used exclusively by females of the subadult and adult class, and exclusively within the context of copulation. The rarer hoo vocalization was only used by adults in our sample, though our *ad libitum* data contains a single recorded instance of a juvenile producing the call, so it may be present in their repertoire at an earlier developmental point. Our sample lacked collected instances of the long call, as it is rarely produced in situations where focal follows are possible. However, our *ad libitum* data collection supports the claim that they are only produced by adult males^60^. Overall, just over half of call types were produced by all age-sex classes (5 out of 9 call types: 55.56% of all call types).

**Fig. 1:**
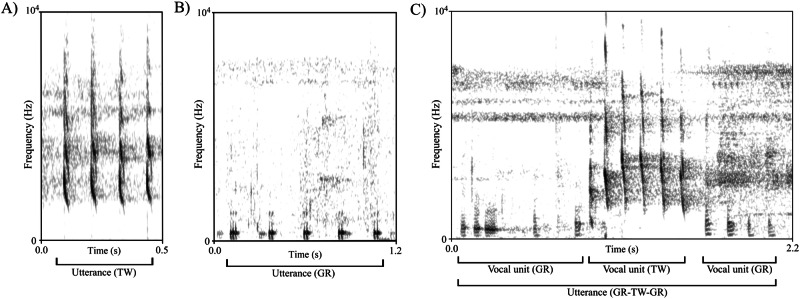
Spectrograms illustrating different types of utterances: single vocal units and vocal sequences. Spectrograms are represented with frequency along the y-axis and time in seconds along the x-axis (**A**) depicts an instance of a single vocal unit/call type twitter (TW) consisting of repetitions of twitter elements, (**B**) depicts an instance of the single vocal unit/call type grunt (GR) consisting of repetitions of grunt elements, and (**C**) depicts a vocal sequence combining the GR and TW vocal units to form a GR-TW-GR sequence. All three examples fall into the overarching category of “utterance” regardless of the number of different vocal units that compose them.

Since we wanted to compare directly the ontogeny of vocal sequences in mangabeys with that found in chimpanzees that live in the same forest, we used the same criteria as in Bortolato et al. 2023^48^ to define a vocal unit and a vocal sequence (Box 1). A vocal unit was made up of a single type of element (e.g., grunt) occurring alone or with repetitions with no more than 2 seconds separating each element (Figure S11). A sequence was defined as the combination of units of different element types (e.g., grunt + twitter) separated by less than 1 second (Figure S12). An utterance is the overarching category that refers to any uninterrupted period of vocalization (following the criteria laid out above) including vocal units produced singly as well as vocal sequences (Fig. 1). The more conservative interval cutoff of 1 second for defining a vocal sequence was chosen as an extra safeguard against over-classification of sequences. Both interval cutoffs fall well below the criteria laid out by Struhsaker^66^, whereby the interval separating units of a given utterance must fall below the interval separating all consecutive utterances (called “bouts” in^66^). To classify each vocal unit, RS used the dual strategy of listening to the recording of each utterance while visually inspecting the spectrogram for the relevant vocal unit features.

In order to validate the 2- and 1-second classification criteria we used, ALF measured 2524 time intervals between elements for 504 utterances produced by adult mangabeys (57% of the adult dataset in our study), 461 of which were single-unit utterances (60% of adult single-unit utterances in our study) and 43 of which were vocal sequences (35% of vocal sequences in our study). The mean ± SE intervals between repetitions of the same element types in a single utterance were 0.25 ± 0.007, and 95% of the intervals were shorter than 0.97 s, both well below the 2 s rule we used. Likewise, the mean ± SE intervals between different vocal units in an utterance were 0.21 ± 0.03, and 95% of intervals were shorter than 0.67 s, both well below the 1 s rule we used. This shows that mangabeys, like chimpanzees and marmosets, are capable of producing different combined sounds in rapid succession. In contrast, the average interval between different utterances within the same audio recording was 6.9 ± 0.52 s—far greater than the intervals encountered within what we defined as utterances.

**Fig. 2:**
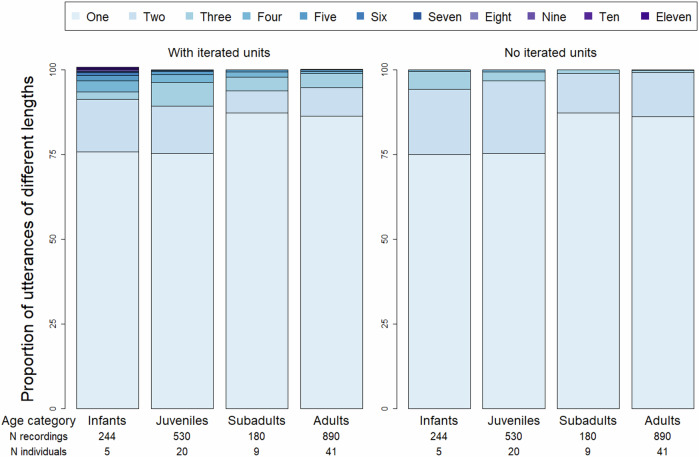
Proportion of sooty mangabey vocal utterances containing 1–11 different call types, categorised by age class. The total number of recordings and individuals making up each age class are listed on the x axis. The gradient of blue indicates sequences of increasing length from lighter to darker. ‘Iterated units’ refers to the repeated emission of a vocal unit that already occurs earlier in the vocal sequence, such that GR-TW-GR is treated as a different sequence to GR-TW. The ‘no iterated units’ condition would remove the repeated instance of GR and treat those two utterances as equivalent.

Overall, vocal sequences represented 18.2% of the utterances produced (335 out of 1844). Utterances with a length greater than three vocal units were extremely rare, comprising only 2.9% of the data set. We recorded 83 different unique vocal sequences but only seven sequences were produced 10 times or more by greater than 3 individuals: GR-TW, GR-TW-GR, TW-GR, VI-GR, WH-TW-WH, TW-SM, and TW-WH (Supplementary Data 1). The vast majority of utterances collected were comprised of only a single vocal unit across all age categories: 75.8%, 75.3%, 87.2% and 86.3% of the utterances produced by infants, juveniles, subadults, and adults, respectively (Fig. 2).

As we could not be sure whether iteration of a vocal unit at a later point in the same vocal sequence (e.g., the second “A” in an A_B_A sequence) adds any information to the utterance, all analyses in our study were conducted in two forms so as to cover both eventualities. The “iterated units included” analyses examined the complete utterance in the form that it occurred, while the “iterated units removed” analyses treated iterations as unimportant and we modified all utterances to exclude them. In the analyses where iterated vocal units have been removed, the overall number of utterances in the sample remains the same but sequences with iterated units have been modified such that GR-TW-GR (Fig. 1c) would be reduced to GR-TW. Subsequent results will therefore indicate whether or not iterated vocal units were included in the analysis.

Box 1 Glossary of terminology *Element*: an element is a continuous sound with a distinct beginning and end. It is the basic constituent of a vocalization event. We discriminate them using their specific acoustic properties (See Supplementary Note 1 for details on the acoustic properties relied upon here).

*Vocal Unit/Call Type*: a vocal unit consists of one or multiple elements of the same type, produced in succession with intervals of less than 2 seconds between each element.

*Vocal Sequence*: a vocal sequence is comprised of consecutive vocal units of different type, where each new vocal unit follows the previous one with an interval of less than 1 second between.

*Utterance***:** an utterance is any vocalization event that can take the form of either a single call produced in isolation (not part of a sequence) or a vocal sequence.

*Utterance Diversity***:** the number of distinct utterances, meaning distinct vocal sequences or vocal units produced singly, produced by an individual or class of individuals.

*Utterance Length***:** the number of different vocal units comprising a particular utterance.

*Iteration***:** the occurrence of a vocal unit multiple times within the same sequence, interspersed with other vocal units. We perform a second analysis throughout our paper in which iterated vocal units are removed.

### Vocal repertoire size with iterated units

When considering all utterance types (i.e., vocal units produced singly and vocal sequences), and vocal sequences with iterated units (i.e., the same vocal units appear several times in the sequences like in Fig. 1c), the data recorded indicated that infants produced a larger diversity of utterances than other age classes, regardless of sex, and that this diversity decreased with age to reach adult levels around the subadult phase of life (Table 1). The larger utterance diversity in infants, however, was primarily driven by the infant-specific call, whimper. When we removed the utterances containing whimpers from our analyses to examine their effect, we found that the difference between age classes was considerably reduced (Figure S13). For all age categories, the mean repertoire size of females was greater than that of males.

**Table 1:** Observed vocal repertoire size and sequence length across sooty mangabey age-sex classes

		Iterated units included		Iterated units excluded	
Age category	Sex	Mean vocal repertoire size (SD)	Mean maximum sequence length (Range)	Mean vocal repertoire size (SD)	Mean maximum vocal sequence length (Range)
Infant	Female	16.0 (-)	9 (9)	14.0 (-)	3 (3)
Juvenile	Female	10.4 (6.8)	4 (3–5)	8.7 (5.5)	2.71 (2–4)
Subadult	Female	6.2 (2.8)	3.4 (2–5)	5.6 (2.4)	2.2 (2–3)
Adult	Female	4.1 (3.0)	2.55 (1–11)	3.7 (2.5)	1.85 (1–3)
Infant	Male	10.2 (2.06)	7.25 (5–11)	9.0 (0.8)	3.25 (3–4)
Juvenile	Male	6.6 (3.3)	2.82 (1–9)	6.3 (3.0)	2.06 (1–4)
Subadult	Male	3.5 (1.9)	2.5 (1–4)	3.0 (1.4)	2 (1–3)
Adult	Male	2.0 (1.2)	1.71 (1–5)	2.0 (1.2)	1.57 (1–4)
Total		5.5 (4.2)	11	5.0 (3.5)	4

Repertoire size refers to the total number of different unique utterances recorded for each individual in the sample, with the above column indicating the mean size and standard deviation for the indicated category. Maximum sequence length indicates the longest vocal sequence recorded out of all individuals in the indicated category. The “iterated units” categories refer to whether iterated vocal units reoccurring within a single utterance were included in the calculation of repertoire size and maximum sequence length.

The age effect on mangabey vocal repertoire size observed in the raw data was confirmed by the results of a Bayesian statistical model that we developed to estimate the vocal repertoire size of each individual mangabey. This model accounts for undersampling of certain utterance types in certain individuals as well as the probability of each call type to be uttered in general and the general propensity of individuals from each age sex class to be more or less vocal. Our approach is fundamentally similar to that of Kershenbaum et al.^67^., but is more flexible and allows for variation in prevalence and rate of production of each unique vocalization, as well as stratification by arbitrary individual and group covariates. We tested this model on simulated data and could show that it recovers well the ground truth vocal repertoire size (Figure S14) even when the observed repertoire size fell well below the ground truth. Please note that the model evaluate the full repertoire size including utterances that we never recorded for certain individuals but that are likely to be present in their repertoire and provides an estimated vocal repertoire size for each individual in the data. Given the rarity of certain utterances the vocal repertoire size estimated by the model is bound to be larger than the observed repertoire.

**Fig. 3:**
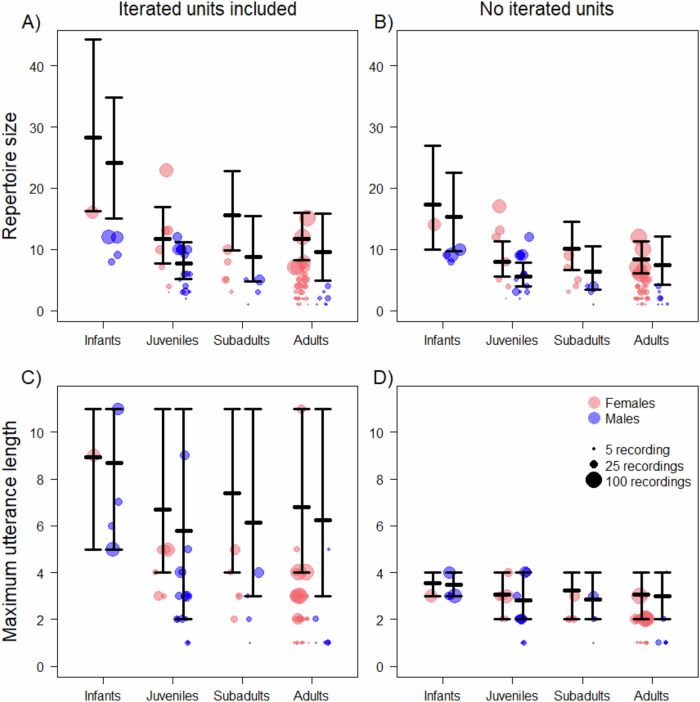
Sex and age effects on mangabey vocal repertoire size and maximum utterance length. **A** Depicts the model predictions presented alongside our raw data for repertoire size (i.e., the number of different utterance types, including both vocal units produced singly and vocal sequences) when iterated vocal units are included, categorised by age class and sex. **B** Presents the same information for the scenario where iterated vocal units have been removed from sequences. **C** Depicts the maximum utterance length with iterated vocal units included and (**D**) presents it with iterated units removed. The thick horizontal black lines indicate the mean posterior prediction derived from the model and the vertical black line the lower and upper bounds of the 89% credible interval. Circles indicate the individuals contributing to our sample, with the size of the circle indicating how many recordings were collected for that individual.

The estimated vocal repertoire sizes derived from the model indicate that infant females consistently produce a larger diversity of utterances than juvenile and adult females and males in all age groups other than infants (contrast comparison between age/sex classes, none of the 89% CI overlapped 0, Table 2). According to the model estimation of full repertoire size, the vocal repertoire of infant females was 141.7%, 81.1% and 140.5% larger than that of juvenile, subadult and adult females respectively (mean estimated repertoire size of 28.23 unique utterances for female infants versus 11.68, 15.59 and 11.74 for female juveniles, subadults, and adults, respectively, Fig. 3a).

**Table 2:** Contrast comparisons between age-sex classes derived from the posterior distribution of the statistical model considering the vocal sequences with iterated units

	Vocal Repertoire Size				Maximum Utterance Length			
Age Class/Sex Comparison*	Mean	SD	Lower 89% CI	Upper 89% CI	Mean	SD	Lower 89% CI	Upper 89% CI
**IF vs. JF**	**16.71**	**9.95**	**2**	**35**	2.32	3.11	−3	7
IF vs. SF	12.65	10.66	−2	33	1.48	3.36	−5	7
**IF vs. AF**	**16.32**	**9.86**	**2**	**36**	2.07	3.39	−4	7
IF vs. IM	4.20	10.85	−13	23	0.25	3.00	−5	6
**IF vs. JM**	**20.36**	**9.57**	**7**	**38**	3.16	3.27	−2	8
**IF vs. SM**	**19.53**	**9.98**	**5**	**38**	2.89	3.31	−2	8
**IF vs. AM**	**18.58**	**10.15**	**4**	**38**	2.79	3.38	−3	8
IM vs. JF	12.51	8.32	−1	26.81	2.07	3.17	−4	7
IM vs. SF	8.45	8.55	−5	23	1.22	3.21	−5	6
**IM vs. AF**	**12.12**	**7.60**	**1**	**25**	1.81	3.29	−5	6
**IM vs. JM**	**16.17**	**7.88**	**5**	**31**	2.90	3.30	−3	8
**IM vs. SM**	**15.33**	**8.19**	**3**	**30**	2.63	3.29	−3	7
**IM vs. AM**	**14.38**	**8.49**	**2**	**29.30**	2.54	3.42	−4	7
JF vs. SF	−4.06	5.92	−14	6	−0.84	3.29	−6	5
JF vs. AF	−0.39	5.16	−9	9	−0.25	3.24	−6	6
JF vs. JM	3.66	4.35	−3	11	0.84	3.46	−5	7
JF vs. SM	2.83	5.50	−7	12	0.57	3.55	−6	7
JF vs. AM	1.87	5.46	−7	11	0.47	3.26	−5	6
JM vs. SF	−7.71	5.43	−18	1	−1.68	3.47	−7	4.81
JM vs. AF	−4.04	4.39	−11	3	−1.09	3.46	−7	5
JM vs. SM	−0.83	4.88	−10	7	−0.27	3.51	−6	6
JM vs. AM	−1.78	4.88	−10	6	−0.37	3.56	−6	6
SF vs. AF	3.67	5.68	−5	14	0.59	3.28	−5	6
SF vs. SM	6.88	6.47	−4	18	1.41	3.41	−5	7
SF vs. AM	5.93	6.11	−3.81	16	1.31	3.51	−5	7
SM vs. AF	−3.21	5.09	−11	5	−0.82	3.61	−7	6
SM vs. AM	−0.95	5.53	−10	8.30	−0.10	3.62	−6	7
AF vs. AM	2.26	5.21	−6	10.30	0.73	3.45	−5	7

Negative estimates indicate that the first comparison group indicated is lower than the second group listed. Bolded rows highlight noteworthy comparisons where the credible interval does not overlap with zero. *IF Infant Female, *JF* Juvenile Female, *SF* Subadult Female, *AF* Adult Female, *IM* Infant Male, *JM* Juvenile Male, *SM* Subadult Male, *AM* Adult Male. Lower and upper 89% CI indicate the lower and upper bonds of the 89% credible interval for each estimate.

Infant males consistently produced a larger diversity of utterances than adult females and all other male age groups (contrast comparison between age/sex classes, none of the 89% CI overlapped 0, Table 2). The vocal repertoire of infant males was 215.1%, 174.6% and 152.5% larger than that of juvenile, subadult and adult males (mean estimated repertoire size of 24.14 unique utterances for male infants versus 7.66, 8.79 and 9.56 for male juveniles, subadults, and adults respectively, Fig. 3A). These age-sex differences were also apparent when inspecting the posterior distribution of estimated vocal repertoire size that was wider and shifted towards larger repertoires in infant males and females than in all the other age-sex classes (Figure S15).

### Vocal repertoire size without iterated units

When removing all iterations of vocal units that appeared previously in the sequence from our recorded vocal sequences (e.g., a sequence A_B_A would become an A_B sequence), the mean repertoire size for all age-sex classes with the exception of adult males marginally declined (Table 1). Removing the vocal sequences with iterated units did not change the effect of age and sex on vocal repertoire size with male and female infants producing a considerably larger diversity of utterances than all other age-sex classes. For all age categories, the mean repertoire size of females was greater than that of the males (Table 1).

In line with the pattern of the empirical data, the estimated vocal repertoire sizes derived from the model indicated the same pattern as for the model with iterated units included (Table S1). According to the model estimation of full repertoire size when iterated units were excluded, the vocal repertoire of infant females was 116.8%, 72.7% and 140.5% larger than that of juvenile, subadult and adult females (mean estimated repertoire size of 17.34 unique utterances for female infants versus 8.0, 10.04 and 8.33 for female juveniles, subadults, and adults, respectively, Fig. 3C). The vocal repertoire of infant males was 173.1%, 140.9% and 105.1% larger than that of juvenile, subadult and adult males (mean estimated repertoire size of 15.32 unique utterances for male infants versus 5.61, 6.36 and 7.47 for male juveniles, subadults, and adults respectively, Fig. 3C).

### Maximum utterance length with iterated units

The distribution of maximum sequence length when considering iterated units indicated a tendency for sequences to be longer in infants than in the other age categories with no obvious differences between the sexes. Yet, the longest utterances (composed of 11 units) were recorded in both infant males and adult females (Table 1), suggesting that age and sex did not have a consistent effect on utterance length across all individuals. As was the case for repertoire size, the tendency for utterance length to be greater in infants was primarily driven by the infant-specific call, whimper, and when this call type was removed from analyses the difference between groups was considerably reduced (Figure S13).

Our statistical models estimated the probability of each utterance type to be present in the vocal repertoire of each age-sex class (Supplementary Data 2) allowing us to simulate potential repertoire for each class. Based on this simulated repertoire, we generated a distribution of maximum utterance length. This analysis confirmed the pattern of the raw data that the mean maximum utterance length tends to be larger in infants (females: 8.92 in infants vs. 6.61, 7.45, and 6.86 in juveniles, subadults, and adults; males: 8.67 in infants vs. 5.77, 6.04, and 6.14 in juveniles, subadults, and adults; Table S2). However, since the estimation of this parameter is associated with large uncertainty and all the 89% CI in each age sex class reach a maximum of 11 units (the maximum recorded in the dataset), the contrast comparison did not indicate consistent difference between age classes or sexes (Table 2, Fig. 3B). When inspecting the posterior distribution of the estimated maximum utterance length based on the simulated repertoire we can still however see that infants have a larger portion of the distribution shifted towards longer maximum utterance (Figure S16).

### Maximum utterance length without iterated units

Exclusion of iterated vocal units resulted in a large reduction in maximum length with a maximum of 4 unique vocal units being combined into a single utterance (Table 1), highlighting the repetitive nature of longer utterances.

Turning to the distribution of maximum utterance length in the simulated repertoire generated by our model (Figs. 3D, S16), the mean maximum utterance length was still greatest in infants, but only by a marginal amount (females: 3.55 in infants vs. 3.06, 3.23, and 3.06 in juveniles, subadults, and adults; males: 3.49 in infants vs. 2.83, 2.84, and 2.98 in juveniles, subadults, and adults; Table S1). Consequently, the contrast comparison as well did not indicate a consistent difference between age classes or sexes with the credible interval of all comparisons overlapping 0 (Table S3). However slight that difference may be, inspection of the posterior distribution of the estimated maximum utterance length reveals that infants are the only age class with the maximum density of the distribution of length occurring at a length of 4, instead of 3 like all other age classes (Figure S14).

## Discussion

Our study investigated the development of vocal sequence complexity in a catarrhine monkey, the sooty mangabey. We computed repertoire size (i.e., the number of different utterance types including single vocal units and vocal sequences) and maximum utterance length estimates across different age-sex classes using a custom-made model designed to account for the probability of each utterance type to be produced and for varying sampling effort across individuals. We found a larger repertoire of vocal sequences and longer maximum vocal sequence length than previously reported in an African monkey. Previous studies typically report less than 10 different vocal sequences containing only a maximum of 2 to 5 vocal units^4,6,11,13,52,65,68,69^ (with the exception of geladas^64^). However, when we removed iterations of the same vocal unit/call type within the same sequence and idiosyncratic sequences, we found a relatively small and fixed vocal repertoire, mainly limited to single calls and bigrams (two-unit sequences), which did not expand across ontogeny. In contrast to recent findings in chimpanzees^4^ and marmosets^21^, in the mangabey, vocal sequence production leaves little room for vocal usage learning of vocal sequences. Rather, sequence production appears to be as genetically fixed as the production of single calls, the latter being a phenomenon already well-described across monkey species^31^. This novel information about fixedness of catarrhine monkey vocal sequence production across ontogeny highlights the potential specificity of the vocal sequence repertoire maturation seen in chimpanzees^48^ and marmosets^21^.

In line with what was previously described in other mangabey species^19,52^, but contrasting with other catarrhine monkeys (e.g., see refs. ^52,65^, see above), when considering also idiosyncratic sequences, we found an exceptionally diverse range of vocal sequences in sooty mangabeys with 90 unique vocal utterance types (9 call types and 83 vocal sequence types). Sequences were not only diverse but also much longer than the commonly described two unit vocal sequences in other monkey species^6,52,65,68,69^, with sequences containing up to 11 vocal units. Adults produced 35 unique utterances when considering iterated vocal units of which 44.4% were unique to that age class (Tables S4, S5, Figures S17, S18). However, when excluding idiosyncratic utterances not produced by at least two individuals and removing iterated units from the utterances, the complete adult repertoire dropped to 21 utterances, of which 14 are bigrams, 1 is a trigram, and the remaining 6 are vocal units produced singly (Figure S19). Furthermore, only 19% of this repertoire is unique to the adult age class. Under these criteria, adult sooty mangabeys had an average repertoire size of 3.2 utterances and a mean maximum utterance length of 1.7 units. This suggests that for the most part, adults only make regular use of a limited portion of their overall repertoire and do not consistently produce sequences above the length of a bigram. This view is consistent with a general trend in non-singing monkey species to emit mainly single calls or short, two-unit vocal sequences, occasionally reaching up to five vocal units^4,6,9,17,52,65,68,69^. So far, only callitrichids (cotton-top tamarins^17^ and marmosets^15,54^) and all four great apes (gorilla^14^, chimpanzee^4,12^, bonobos^7,70^ and orangutan^71,72^) were reported to have a large repertoire of vocal sequences. The combination of individually-produced vocal units into short vocal sequences has been documented outside of the primate order as well in birds^73,74^, elephants^75^, bats^76,77^, and mongooses^59,78,79^.

When analysing the developmental pattern of vocal sequence production we found no support for protracted development throughout ontogeny, although protracted acquisition has been demonstrated in marmosets^21^ and chimpanzees^48^. Chimpanzees show a clear increase in their repertoire of vocal sequences and utterance length across the first decade of life. In chimpanzees, the relationships between utterance diversity and event diversity as well as between utterance length and number of concomitant events increase with age^48,80^, suggesting the possibility that, generally, different utterances (including vocal sequences) convey different meanings and that longer utterances convey more than one meaning. In stark contrast, infant sooty mangabeys actually produced a larger diversity of vocal sequences than older individuals, and tended to produce longer or similar-length vocal sequences. These results support our first hypothesis that unlike apes, the repertoire of vocal sequences is mostly fixed from birth in catarrhine monkeys. Importantly, whereas the innate fixedness of monkey vocal repertoires is well established when it comes to single calls, we provide the first evidence that this inflexibility also applies to the production of monkey vocal sequences. Comparisons of vocal sequence structure across populations, as conducted in chimpanzees^46^, may be useful to confirm that, like single calls, vocal sequences are innate species-specific traits not subject to vocal learning in mangabeys.

With the exception of a few age-specific single vocal units, such as the whimpers produced only by infants and juveniles and the vibrato produced only by sexually mature females, the bulk of sooty mangabey single vocal units are produced soon after birth and remain relatively stable throughout life. When considering vocal sequences, infant mangabeys had a larger overall vocal repertoire of different utterance types as compared to other age classes. We did not predict that the level of vocal complexity observed in infants would outstrip that of the other age classes. However, it is important to note that many of these sequences (34.9%) consisted of iterations of two units several times (often involving the infant-specific “whimper” vocalization) to form long sequences comprising up to 11 units. When considering vocal sequences without iterated units, infants still had a larger vocal repertoire but the difference with other age classes was less pronounced and the difference in maximum utterance length disappeared.

Importantly, not only did we not find an expansion of the vocal repertoire with age, we additionally found that the repertoire of vocal sequences used by infants and juveniles already comprised most of the adult vocal sequences that do not contain vocal units that are functionally unique to adults. In fact, when considering vocal sequences produced by at least two individuals in our sample (to exclude potentially idiosyncratic sequences) and when excluding sequences containing call types that are associated with adult-specific behaviours (e.g. copulation-related calls) we found that 86.4% of the adult vocal repertoire was produced by either infants or juveniles (Figure S20). This provides further support for the hypothesis that vocal sequence production in mangabeys is mostly innate and present soon after birth. However, the fact that the infants in our data set tended to have more diverse utterance sets when compared to other age groups can stem from two non-mutually-exclusive phenomena.

One possibility is that of baby babbling^81^, and though the pattern observed in our data is superficially suggestive of this, there are certain key features that are lacking for the infant utterances to meet this definition. For one, the chattering characteristic of the infant’s long utterances is produced primarily in response to maternal separation or rejection (personal observation). Additionally, 68.8% of lengthy utterances (those with a length > = 5) produced by infants and juveniles involved the vocal unit whimper, which is a call type unique to the infant and juvenile age groups, supporting the idea that long infant vocal sequences are emitted to attract maternal attention and do not represent adult repertoire experimentation. Exploratory removal of vocal sequences containing whimpers largely eliminated the differences we observed between infants and other age classes (Figure S13), illustrating that their predominance in the infant repertoire accounts for the between-age differences that we observed. Interestingly, infants almost systematically combined whimpers with other call types (Figure S10) and, unlike juveniles, rarely produced whimpers alone. It is therefore more likely that the lengthy calls used by younger individuals represent infant distress calls. Such calls are widespread across vertebrates exhibiting parental care^82^, including species as diverse as elephants^83^, chickens^84^, and crocodiles^85^. They are also present across the primate order^86,87^ and in many cases involve the production of multiple call types (rhesus macaque^88^; guinea baboons^89^; yellow baboons^90^; tufted capuchins^91^; pygmy marmoset^92^; common marmoset^88,93^; bush babies^94^). Typical features are their high pitch components and lengthy production^82,87^. They are most consistently produced when infants are separated from their mother^86,87^. The acoustic features and behavioural contexts associated with distress calls are consistent with the profile of the lengthy utterances produced by the infant (and weaning-age juvenile) sooty mangabeys in our study. Because the specific composition of such bouts varied considerably from episode to episode, it resulted in individuals of this age group having a large vocal repertoire. The lengthy nature of these distress calls additionally resulted in infants achieving a high maximum utterance length relative to most other age groups.

In conclusion, we found no increase in vocal sequence length or diversity with age in sooty mangabeys, supporting the view that the monkey vocal repertoire of single calls and vocal sequences emerges shortly after birth and is largely stable across the lifespan. We documented the production of all single vocal units within the first two years of life in mangabeys, with the exception of two complex calls related to adult-specific behaviours, which did not emerge until sexual maturity. The overlap between the vocal sequence repertoire of all age classes was high, suggesting that developmental processes played little role in its emergence and excepting those sequences involving vocalizations associated with adult-specific behaviours, the vocal sequence repertoire does not greatly change across the lifespan. We additionally demonstrated that infants produce a greater diversity of vocal sequences than all other age classes. We attributed this to their frequent production of distress calls, which tended to be lengthy in nature and involve many iterated vocal units. This divergence from an otherwise stable usage pattern in no way contradicts our vision of a fixed vocal repertoire in the species, but instead represents a form of vocal production particular to the needs of infants.

Our overall conclusion is in concert with the image of a fixed vocal repertoire in primate species^31^, suggesting that the shared developmental pattern observed in chimpanzees^48,80^ and marmosets^21^ may be an instance of convergent evolution. We propose that the protracted vocal repertoire development observed in chimpanzees constitutes a derived, rather than ancestral, state, with the pattern observed in sooty mangabeys being more representative of the pattern characteristic of catarrhine monkeys. Assessing whether the proposed derived state observed in chimpanzees constitutes a pattern typical of great apes or of monkeys with cooperative breeding systems like marmosets requires similar examination of other monkey and ape species. This would be important to establish for theories of language evolution, particularly as they pertain to cognitive expansion and its relation to structural changes in relevant brain areas through the hominid lineage. Vocal production learning has been documented in a wide range of taxa including songbirds^95^, bats^96^, pinnipeds^97^, and cetaceans^98^ but within primates, humans appear uniquely capable of this ability. Yet, language is not only the capacity to acquire new sounds, but the ability to recombine them into words and sentences. The human capacity to learn vocal combination is possibly shared with great apes and with the more distantly related marmosets. It is therefore surprising that we did not find evidence of such a possibility in the mangabey, a catarrhine monkey with a relatively rich vocal sequence repertoire, and more closely related to us than marmosets, suggesting that this trait evolved independently in the great ape and the Platyrrhine lineage. It is however not out of keeping with the general lack of evidence for vocal learning in catarrhine monkeys^99,100^.

## Methods

### Study sites and subjects

We conducted the study with two groups of wild sooty mangabeys fully habituated to human observers, the Taï Chimpanzee Project group (TCP^101^) and the Taï Monkey Project group (TMP^57^) in the Taï National Park, Ivory Coast. Taï National Park is a primary rainforest environment with an average temperature of 24°C and an average rainfall per year of 1800mm. The permanent field sites of the TCP and TMP are designed to have minimal impact on the surrounding environment. We collected data from November 2020 to April 2021 and from March to July 2022 on the TCP group and from May to July 2022 on the TMP group.

The individuals included in our sample were of diverse age (estimated age from 1 month to 16 years) so as to provide a representative cross-section of the mangabey cycle. We classified individuals into four age classes: infants, juveniles, subadults, and adults. This judgement was based on age of the individual, when known, and knowledge of developmental milestones, when unknown (details available in Supplementary Note 1). All infants, juveniles, and subadults of known age were included in the sample. Because adults were overrepresented in our study groups, we randomly selected a subset of them to build our sample so as to have a more comparable sample size to our other age groups. During our period of data collection, the TCP group consisted of 56 individuals, 28 females and 28 males, while the TMP group consisted of approximatively 59 individuals (sample breakdown in Table S6).

### Data collection

RS, ALF and TSA conducted continuous focal follows^102^ of 4.5 consecutive hours for adults and 1 h for all other age groups. During this period, audio recordings were collected using a Seinnheiser directional microphone (models used were MKH 416 P48, MKH 316 and K6) connected to a Marantz 661 solid-state recorder (44.1 kHz sampling rate, 16 bits accuracy, WAV format). All authors participating in data collection succeeded in an inter-observer reliability test for mangabey identity recognition before beginning data collection. We collected a total of 784.6 hours of focal follow from 75 individuals (34 adult females, 7 adult males, 5 subadult females, 4 subadult males, 6 juvenile females, 14 juvenile males, 1 infant female, and 4 infant males).

In total, we collected 1874 utterances, 236 of which had been recorded by a research assistant during a previous field season using the same equipment as in our study. Only utterances recorded in their entirety from known callers were included in the sample. A total of 30 utterances were too indistinct to be reliably identified and were excluded from the sample, leaving a total of 1844 utterances in our final sample (Table S6). All 75 study subjects were included in our final sample, some of which were included in more than one age class due to data collection occurring over two field seasons. Overall, our sample comprised 244 utterances from 5 infants, 530 utterances from 20 juveniles, 180 utterances from 9 subadults, and 890 utterances from 41 adults (Table S6).

### Processing and analysis of recordings

We examined all utterances in our sample using PRAAT software^103^, which displays spectrograms showing frequency distribution over time. The calls in the sooty mangabey vocal repertoire have distinctive profiles that are readily visible in their spectrogram representation (see Figures S1−S9 and ref. ^60^). To classify the vocal units in an utterance, RS listened to the recording of the utterance while visually inspecting the spectrogram for the relevant vocal unit features. In the event that any vocal unit in an utterance could not be positively identified using this method, the utterance was excluded from the sample.

In order to test inter-rater agreement of utterance classification, ALF independently coded 291 utterances comprised of 390 vocal units, which accounted for 15.8% of our overall sample. These utterances were selected randomly with the constraint that they were split evenly across all age classes. We tested inter-rater agreement at two different levels: agreement on the vocal units composing each utterance and a judgement of whether an utterance was composed of only a single call type or constituted a vocal sequence. Agreement was moderate in the case of both vocal unit identification (Cohen’s kappa = 0.78) and the singly vs. sequence judgement (Cohen’s kappa = 0.72), according to the interpretation standards suggested by McHugh^104^. As we expected to achieve higher inter-rater scores, we examined whether they were attributable to ratings of any particular age class or call type. We found that grunts in particular had a low inter-rater score, especially in non-adult age classes. This call type is often subtle when produced by younger individuals, and thus can be occasionally overlooked during the coding process. However, any under-identification of grunts that may have occurred evidently did not affect the overall pattern we observed: occasionally overlooking grunts should have, if anything, resulted in shorter sequences and lower repertoire diversity for the affected class, but the youngest individuals in our study instead tended to have higher sequence length and diversity than the adults. When grunts were removed from the test, agreement scores increased to 0.83 for vocal unit identification and 0.85 for the singly vs. sequence judgement.

### Analyses

We used two primary measures of vocal complexity in our analyses, vocal repertoire size and maximum utterance length. Following Bortolato et al.^48^., vocal repertoire size represents utterance diversity, calculated as the total number of unique utterances produced by each individual, including vocal units produced singly and vocal sequences. Maximum utterance length represented the number of vocal units in the longest vocal sequence produced by each individual. Both measures were examined with and without iterated instances of the same vocal unit occurring within the utterance, such that in the non-iterated case, the utterance GR-TW-GR of length three (Fig. 1C) would simplify to GR-TW of length two. The analyses removing iterated units were carried out to account for the possibility that iterated instances of the same vocal unit within a sequence represent repetitions of essentially the same message and, from the perspective of the mangabeys, do not represent unique utterances. In order to exclude potentially idiosyncratic calls not belonging to the typical repertoire of sooty mangabeys, we included a final, more conservative case, in which utterances that had not been produced by at least two individuals were excluded from the data set (Figure S21).

All these analyses were iterated with the exclusion of complex calls (Figure S22) as well as with the subadults merged into the adult class (Figure S23) to determine whether this would alter the observed pattern and ease comparability with other studies. All statistical analyses were conducted using R (Version 4.3.1) software.

### Statistics and reproducibility

We developed a model to provide a more accurate estimate of the full repertoire of the individuals in our study. The primary estimand is the vocal repertoire of each individual. The primary problem in estimating this repertoire is that a finite sample will tend to underestimate the total repertoire. This is especially true if some vocalizations are used rarely. This problem has been recognized specifically in vocal repertoires for decades, and is shared with diversity estimation more generally^67,105,106^. We build upon previous work by constructing a new probabilistic estimator of individual repertoire that accounts for undersampling.

Specifically, let *M* be the number of unique vocalizations in the population. Each of *N* individuals has an unknown repertoire *phi*, which is an *M*-length vector of zeros and ones, indicating which vocalizations the individual possesses. When an individual is observed to produce vocalization *m*, then *phi*_*m* is assigned 1. The estimation comes when *m* is not observed in the sample. Assume that vocalization *m* is produced at a rate *lambda*_*m*, when *phi*_*m* = 1. Then in a recording of duration *d*, the observed count of vocalization *m*, *Y*_*m*, is Poisson distributed with mean *d lambda*_*m*. For *Y*_*m* > 0, the probability of the data is given by the Poisson probability mass function

$$\Pr \left({Y\_m}=y\left|{lambda\_m},d,{p\_m}\right.\right)={p\_m}{\left({lambda\_m}\,d\right)}^{\wedge }{y}\exp \left({{{-}}}{lambda\_m}d\right)/y!$$

where *p_m* is the unknown prevalence of vocalization *m* in the population. When Y_m = 0, we require instead a mixture probability accounting for the possibility that *phi*_*m* = 1 but that *m* nevertheless went unobserved. This probability is given by:

$$\Pr (Y{{\_}}m = 	\, 0\left|{lambda}{{\_}}m,d,p{{\_}}m\right.)=p{{\_}}m{{\rm{Pr}}}(Y{{\_}}m=0\left|{phi}{{\_}}m=1\right.) \\ 	+(1-p{{\_}}m)\Pr (Y{{\_}}m=0\left|{phi}{{\_}}m=0\right.)=p{{\_}}m\,{\mathrm{exp}}({{-}}{lambda}{{\_}}m\,d)+(1{{{-}}}p{{\_}}m)$$

This probability expression allows us to compute the probability that an individual possesses *m*, Pr(phi_m = 1), given *m* was not observed. By Bayes theorem:

$$\Pr ({phi}{{\_}}m=1)=\Pr (Y{{\_}}m=0\left|{phi}{{\_}}m=1\right.)\Pr ({phi}{{\_}}m=1)/\Pr (Y{{\_}}m=0\left|{lambda}{{\_}}m,d,p{{\_}}m\right.)$$

We implemented this model in the Stan probabilistic programming language^107^. We stratified the variables *lambda* and *p* by age category (infant, juvenile, subadult, adult) and sex, but this is not an essential feature of the approach and is easily modified in the provided code. The detailed sample size for the data used in the analyses and in particular the number of individuals of each sex and age class as well as the number of utterances analysed are all presented in Table S6. We validated our implementation by simulating synthetic samples of vocalizations in which ground truth repertoires are known and verifying that the code functions as intended. In particular, we tested the ability of our model to recover ground truth on a simulated dataset of 50 individuals in a theoretical species with 50 different utterance types and with 20 recordings per individuals. This test led to accurate recovering of ground truth even for individuals where the observed vocal repertoire size fell far below the true repertoire size (see Figure S14 and details at https://github.com/rmcelreath/cg_vocal_repertoires/blob/main/simulation.r). In addition to the Stan code, we provide an implementation using the rethinking package, which may be more convenient to modify^108^. The code uses Hamiltonian Monte Carlo to produce samples from the posterior distributions for the repertoire of each individual. All the codes can be found alongside the dataset in the code repository (https://github.com/tozbu/mangabey_voc_sequence). We employed standard MCMC diagnostics as explained in^109^. We ran three chains for 500 samples and computed R_hat and bulk effective sample size for all variables. All chains appear to have converged to the target posterior distribution, as R_hat converged to 1 and bulk effective sample size was high for all variables.

### Ethics

Data collection protocol was observational and non-invasive, approved by the ‘Ethikrat der Max-Planck Gesellschaft’, allowed by the Ministère de l’Enseignement supérieur et de la Recherche scientifique; des Eaux et Forêts en Côte d’Ivoire and the Office Ivoirien des Parcs et Réserves. We have complied with all relevant ethical regulations for animal use.

### Reporting summary

Further information on research design is available in the Nature Portfolio Reporting Summary linked to this article.

## Supplementary information

Supplementary Information

Supplementary Data 1

Supplementary Data 2

Reporting Summary

Description of Additional Supplementary Files

## Data Availability

The data used for this study are available in the repository (https://github.com/tozbu/mangabey_voc_sequence) and 10.5281/zenodo.14900576.

## Code availability

The R and Stan codes used for this study are available in the repositories (https://github.com/tozbu/mangabey_voc_sequence) and 10.5281/zenodo.14900576.
