# Supplementary material for: Vocal sequence diversity and length remain stable across ontogeny in a catarrhine monkey (Cercocebus atys)
Source: Commun Biol. 2025 Mar 20;8:465. doi: 10.1038/s42003-025-07922-2 (PMC11926236; doi:10.1038/s42003-025-07922-2)
Supplement: Supplementary file 1 — Supplementary Information [file 42003_2025_7922_MOESM1_ESM.pdf]

Supplementary Materials

Supplementary Note 1. Age class designation additional details

Age class designation explanation

In the TCP group, we used the genealogy from our long-term data to determine the exact age of individuals at the time of sampling, where possible. In cases of known age, all individuals under the age of 1 were classified as infants. Females were classified as juveniles from the age of 1-4, subadults from 4-5, and adults beyond the age of 5. Males were classified as juveniles from the age of 1-5, however, as male migration occurs between groups from around the age of puberty onwards (2), the exact age of individuals beyond this developmental milestone was not known. Additionally, the exact ages of focal individuals in the TMP group were not known. In cases of unknown age, individuals are classified as infants until they reach weaning age and gain full independence from their mother. From the age of weaning until the onset of sexual maturity, individuals are classified as juveniles. Subadults are sexually mature individuals who have not yet reached their full adult musculature, at which point they are classified as adults.

Mangabey life cycle details in support of age class designations

In the first year of life, mangabeys are nutritionally dependent on their mothers with a high frequency of preferential interaction occurring between them (3). Mangabeys are weaned and achieve nutritional independence from their mother around the age of 1 (4), marking their transition from the infant to the juvenile phase of life. Individuals that were nutritionally dependent on their mother or that were known to be under the age of 1 were thus marked as infants.

A diverse set of markers varying by sex and largely centered around achievement of sexual maturity define the transition from juvenility to subadulthood. In mangabeys, both sexes and all age groups, excluding infants, have been recorded to show high rates of sexual behaviour including erection and intromission (5). The presence of such behaviour then constitutes a poor measure of sexual maturity, as females are not capable of conceiving until later in life. This is evidenced by the fact that the average age of first birth is 4.66 years in captivity (6) and 5.9 years of age in the wild population we have had under observation. Female mangabeys in captivity experience their first sexual swelling around the age of 3 (6,7), though the average age may be slightly higher in wild populations as the youngest female in our sample observed with a sexual swelling was 4.5 years of age. We thus defined the female subadulthood period as commencing at the age of first sexual swelling and continuing to either the age of first birth or

the achievement of full adult musculature. In the case of females of known age, we marked the subadult period as occurring from the age of 4 to 5, which generally corresponds to the age of first swelling while preceding the earliest age at which birth has been observed in our wild population. Females who were either over the age of 5, had already given birth, or had clearly achieved their full adult musculature were thus marked as adults.

An indication of sexual maturity in males is the heightened serum testosterone levels and associated phase of rapid body growth occurring between 55 and 72 months (8). This corresponds to the period where males achieve rank independence from their mother with them coming to outrank most females when they are between the age of 4 and 7 (4). Captive studies have shown that males are of the same approximate weight as females until around the age of 5, which roughly corresponds to the onset of the rapid growth period in males (8) and the age we have marked as adulthood in females. The onset of this hormonal shift in males could thus be inferred through their surpassing adult female size. Therefore, males who were at least the size of an adult female but had not yet reached their full adult musculature were classified as subadults, beyond which time they were classified as adult. The age of 5 could thus be set as the approximate age where males transition from juvenility to subadulthood, though this was irrelevant to our study as there were no males of known age beyond that point. Males were marked as adult once they had achieved their full adult musculature, signaling the end of their period of their rapid growth/elevated testosterone period.

Supplementary tables

Table S1. Main model results with iterated units removed

		Vocal Repertoire Size				Maximum Sequence Length			
Age	Sex	Mean	SD	Lower 89% CI	Upper 89% CI	Mean	SD	Lower 89% CI	Upper 89% CI
I	F	17.33828	5.451101	9.952187	26.99553	3.545333	0.531967	3	4
J	F	7.995902	1.821852	5.55583	11.28948	3.056	0.673401	2	4
S	F	10.03795	2.590133	6.610108	14.45254	3.226667	0.613017	2	4
A	F	8.332558	1.720704	6.023225	11.30474	3.056	0.651227	2	4
I	M	15.32213	4.039591	9.6656	22.52927	3.486667	0.543645	3	4
J	M	5.610482	1.202308	3.979622	7.762431	2.825333	0.752494	2	4
S	M	6.362961	2.352041	3.389779	10.53207	2.844	0.767104	2	4
A	M	7.468158	2.692732	4.167838	12.15919	2.978667	0.735935	2	4

The left-most age column indicates age class: I = infant, J = juvenile, S = subadult, A = adult. In the sex column F = female and M = male. The mean columns refer to the mean of the posterior distribution for that measure. SD indicates the standard deviation, with the CI columns indicating the upper and lower boundaries of the 89% confidence interval.

Table S2. Model estimates of the repertoire size and maximum sequence length with iterated vocal units included.

		Vocal Repertoire Size				Maximum Utterance Length			
Age	Sex	Mean	SD	Lower 89% CI	Upper 89% CI	Mean	SD	Lower 89% CI	Upper 89% CI
Infant	Female	28.23	8.82	16.30	44.33	8.92	2.28	5.00	11.00
Juvenile	Female	11.68	3.08	7.63	16.92	6.61	2.57	4.00	11.00
Subadult	Female	15.59	4.21	9.88	22.82	7.45	2.68	4.00	11.00
Adult	Female	11.74	2.49	8.23	16.04	6.86	2.69	4.00	11.00
Infant	Male	24.14	6.41	15.01	34.90	8.67	2.34	5.00	11.00
Juvenile	Male	7.66	1.97	5.11	11.20	5.77	2.67	2.00	11.00
Subadult	Male	8.79	3.36	4.79	15.43	6.04	2.68	3.00	11.00
Adult	Male	9.56	3.57	4.93	15.90	6.14	2.75	3.00	11.00

The mean columns refer to the mean of the posterior distribution for that measure. SD indicates the standard deviation, with the CI columns indicating the upper and lower boundaries of the 89% confidence interval. Female results are indicated in pink and male results in dark blue.

Table S3. Contrast comparisons for model with iterated vocal units removed

Vocal Repertoire				Max Length				Age Comparison	
Mean	SD	Lower 89% CI	Upper 89% CI	Mean	SD	Lower 89% CI	Upper 89% CI	age.sex1	age.sex2
-9.23067	6.480853	**-21**	**0**	-0.48933	0.808347	-2	1	**J_F**	**I_F**
-7.18133	6.746144	-19	3	-0.31867	0.786093	-2	1	S_F	I_F
-8.62667	6.369515	**-20**	**0**	-0.48933	0.800046	-2	1	**A_F**	**I_F**
-1.644	7.300159	-14.295	9	-0.05867	0.73753	-1	1	I_M	I_F
-11.3893	6.32902	**-23**	**-2**	-0.72	0.865601	-2	1	**J_M**	**I_F**
-10.7347	6.716677	**-24**	**-1**	-0.70133	0.885349	-2	1	**S_M**	**I_F**
-9.51067	6.886008	**-22**	**0**	-0.56667	0.86016	-2	1	**A_M**	**I_F**
2.049333	4.040049	-4.295	8	0.170667	0.860151	-1	2	S_F	J_F

0.604	3.633288	-6	7	0	0.884521	-1	1	A_F	J_F
7.586667	5.350451	-1	16.805	0.430667	0.800446	-1	2	I_M	J_F
-2.15867	3.475283	-8	3	-0.23067	0.939487	-2	1	J_M	J_F
-1.504	4.116055	-9	5	-0.212	0.982689	-2	1	S_M	J_F
-0.28	4.293193	-7	7	-0.07733	0.939786	-2	1	A_M	J_F
-1.44533	3.978792	-8.295	5	-0.17067	0.852355	-2	1	A_F	S_F
5.537333	5.533302	-3	14.805	0.26	0.761472	-1	1	I_M	S_F
-4.208	3.880311	-11	2	-0.40133	0.923075	-2	1	J_M	S_F
-3.55333	4.306881	-11	3	-0.38267	0.922334	-2	1	S_M	S_F
-2.32933	4.54875	-10	5	-0.248	0.919941	-2	1	A_M	S_F
6.982667	5.141363	-1	15	0.430667	0.793746	-1	2	I_M	A_F
-2.76267	3.309523	-9	2	-0.23067	0.919377	-2	1	J_M	A_F
-2.108	3.729909	-8	4	-0.212	0.967628	-2	1	S_M	A_F
-0.884	4.052543	-7	6	-0.07733	0.918229	-2	1	A_M	A_F
-9.74533	5.082019	**-19**	**-2**	-0.66133	0.88148	-2	1	**J_M**	**I_M**
-9.09067	5.47818	**-19**	**-0.195**	-0.64267	0.933171	-2	1	**S_M**	**I_M**
-7.86667	5.663537	**-18**	**0**	-0.508	0.879565	-2	1	**A_M**	**I_M**
0.654667	3.660776	-5	6	0.018667	1.028135	-2	2	S_M	J_M
1.878667	4.071451	-5	9	0.153333	1.000245	-2	2	A_M	J_M
1.224	4.143998	-5	8	0.134667	1.020091	-2	2	A_M	S_M

Negative estimates indicate that the first comparison group indicated is lower than the second group listed. Bolded rows highlight noteworthy comparisons where the credible interval does not overlap with zero. *: IF = Infant Female, JF = Juvenile Female, SF = Subadult Female, AF = Adult Female, IM = Infant Male, JM = Juvenile Male, SM = Subadult Male, AM = Adult Male. Lower and upper 89% CI indicate the lower and upper bonds of the 89% credible interval for each estimate.

Table S4. Proportion of utterances in the vocal repertoire of each age class unique to that age class compared to each other age class

	Total utterances	Unique from infants	Unique from juveniles	Unique from subadults	Unique from adults	Total unique
Infants	32	-	19 (59.4%)	24 (75%)	22 (68.8%)	18 (56.3%)
Juveniles	50	37 (74%)	-	39 (78%)	34 (68%)	30 (60%)
Subadults	16	8 (50%)	5 (31.3%)	-	3 (18.8%)	3 (18.8%)
Adults	35	25 (71.4%)	19 (54.3%)	22 (62.9%)	-	16 (44.4%)

The “total utterances” column indicates the total unique utterances in our sample for that age class. The “unique from…” columns indicate how many of those utterances are not present in the utterance set of the indicated comparison groups (proportion given in brackets).

Table S5. Complete vocal repertoire of the sooty mangabey demonstrating presence of each utterance by age group (with iterated vocal units included).

Call name	Infant presence	Juveniles presence	Subadult presence	Adult presence
GR_TW	1	1	1	1
GR	1	1	1	1
TW	1	1	1	1
TW_GR	1	1	1	1
SM	1	1	1	1
TW_GR_TW_GR	1	1	1	1
GO	1	1	1	1
GR_TW_GR	1	1	1	1
GR_TW_GR_TW_GR	0	1	1	1
SC	0	1	1	1
TW_GO	0	1	1	1
VI	0	0	1	1
SM_GO	0	0	1	1
TW_SM	1	1	0	1
TW_GR_TW	0	1	0	1
SM_GO_SM	0	1	0	1
GR_TW_GR_TW	0	1	0	1
SC_TW	0	1	0	1
SM_GR	1	0	0	1
SC_GR	0	0	0	1
GO_TW_GO_SM_GO	0	0	0	1
GO_TW_GO	0	0	0	1
VI_SM_GR	0	0	0	1
VI_GR	0	0	0	1
GR_VI	0	0	0	1
GO_SM	0	0	0	1
GR_SM_HO_GO_SM	0	0	0	1
GR_GO_GR	0	0	0	1
HO_GR	0	0	0	1
VI_GO	0	0	0	1
SC_GR_TW_GR	0	0	0	1
GR_TW_HO_GR	0	0	0	1
GR_SC	0	0	0	1
GO_GR_GO_GR_GO_GR_GO_GR_GO_GR_GO	0	0	0	1
GR_TW_GR_GO	0	0	0	1
GR_TW_GO_GR	0	0	1	0
GR_TW_GO	0	0	1	0

GO_GR	0	0	1	0
WH	1	1	0	0
TW_WH	1	1	0	0
WH_TW	1	1	0	0
SM_WH	1	1	0	0
WH_GR_TW	0	1	0	0
GR_TW_WH	0	1	0	0
WH_TW_WH	0	1	0	0
WH_SM_WH	0	1	0	0
GO_TW	0	1	0	0
WH_GR	0	1	0	0
WH_TW_GR	0	1	0	0
WH_TW_SM_TW_WH	0	1	0	0
WH_TW_SM_TW_SM	0	1	0	0
TW_WH_TW_WH	0	1	0	0
WH_TW_WH_TW	0	1	0	0
WH_SM	0	1	0	0
TW_GR_TW_GO	0	1	0	0
TW_GO_TW_GO	0	1	0	0
SM_TW	0	1	0	0
SM_TW_GO_TW	0	1	0	0
WH_SM_TW_WH_TW	0	1	0	0
SM_TW_GR	0	1	0	0
WH_SM_TW_GR	0	1	0	0
SM_GO_TW	0	1	0	0
GR_WH	0	1	0	0
WH_TW_SM_GO_TW_WH	0	1	0	0
WH_TW_GR_WH	0	1	0	0
WH_TW_WH_TW_WH_TW_GO_TW_GO	0	1	0	0
GR_GO	0	1	0	0
GR_SM	0	1	0	0
TW_SM_SC_WH	0	1	0	0
WH_TW_WH_GR	0	1	0	0
TW_SM_TW_GR	0	1	0	0
TW_SM_TW	0	1	0	0
TW_SM_TW_SM	1	0	0	0
SM_WH_SM_WH_SM	1	0	0	0
SM_WH_SM_TW	1	0	0	0
SM_WH_SM	1	0	0	0
SM_WH_TW_WH_TW_WH_SM_WH_SM_TW_WH	1	0	0	0
TW_SM_WH_TW_WH	1	0	0	0

GR_TW_SM_TW_GR_TW_SM_WH_TW_WH	1	0	0	0
SM_GO_SM_GO_SM_GO	1	0	0	0
GR_TW_SM_TW_GR	1	0	0	0
GR_SM_TW_GR	1	0	0	0
GR_TW_SM_TW_SM_GR	1	0	0	0
WH_TW_WH_TW_GR_TW_WH	1	0	0	0
TW_WH_TW_WH_TW_SM_TW_WH_TW	1	0	0	0
SM_TW_SM_WH	1	0	0	0
SM_TW_WH	1	0	0	0
SM_SC	1	0	0	0
TW_SM_TW_WH	1	0	0	0
TW_SM_GO_TW_GO	1	0	0	0

Utterances are listed in the left-most column with the two-letter short forms for each vocal unit making up the utterance separated by "_" from subsequent units in that utterance. The columns to the right indicate which age classes each utterance was present in, with "1" indicating presence and "0" indicating absence. GR = growl, TW = twitter, GO = growl, SM = scream, WH = whimper, VI = vibrato, HO = hoo, SC = shrill call

Table S6. Vocal sample breakdown by age category and sex

Age category	Sex	Number of individuals	Number of utterances	Mean utterances per individual (min - max)	Single unit utterances	Vocal sequences
Infant	Female	1	61	61.0 (-)	47	14
Juvenile	Female	6	203	33.8 (3 - 73)	130	73
Subadult	Female	5	108	21.6 (10 - 40)	93	15
Adult	Female	34	833	24.5 (1 - 105)	715	118
Infant	Male	4	183	45.8 (21 - 82)	138	45
Juvenile	Male	14	327	23.4 (1 - 48)	269	58
Subadult	Male	4	72	18.0 (3 - 45)	64	8
Adult	Male	7	57	8.1 (3 - 16)	53	4
Total		75	1844	24.6 (1 - 105)	1509	335

Age class and sex of the described group are indicated in the two left-most columns. Column totals are given in the bottom row. Vocal sequences are utterances with more than one vocal unit.

Supplementary figures

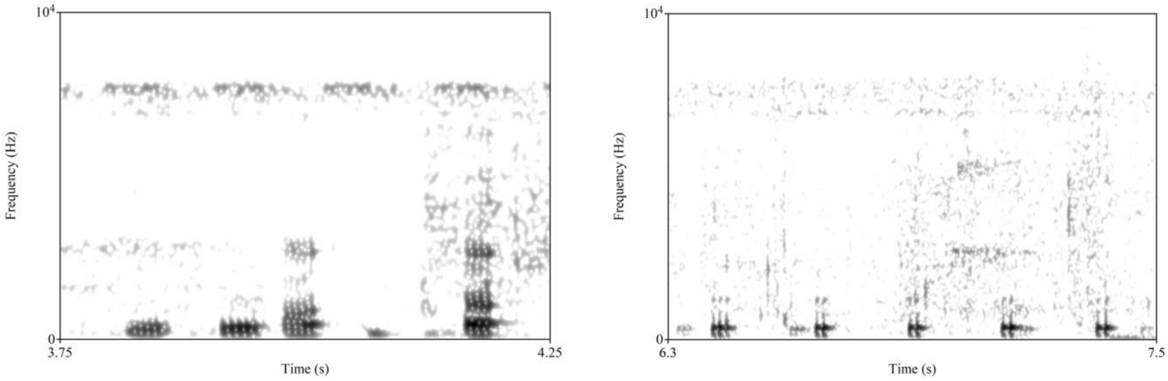

Figure S1. Spectrogram representation of a Grunt (GR) vocalization.

Grunts are low-frequency vocalizations that are produced in a wide range of contexts. They are invariably short (<200 milliseconds) and often produced with many repetitions. Grunts are used by all age groups, are frequently included in vocal sequences, and are the most common vocal unit produced singly by adults and subadults (**Figure S11**).

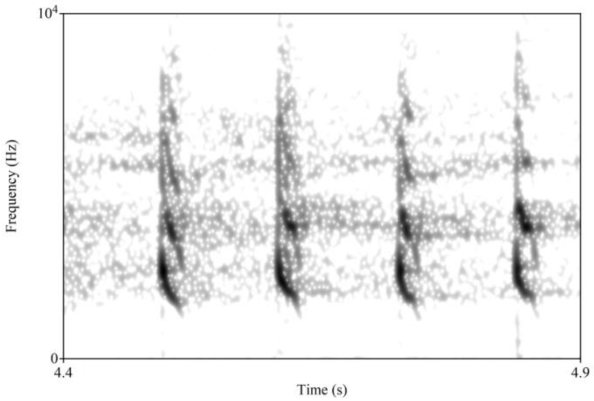

Figure S2. Spectrogram representation of a Twitter (TW) vocalization.

Twitters are short, high-frequency vocal units with a fundamental frequency around 1 kHz and many visible overtones extending up to 20 kHz. Twitters are used by all age groups, are frequently included in vocal sequences, and are the most common vocal unit produced singly by infants (**Figure S11**). Though used by adult females, adult males have never been observed to produce this vocal unit.

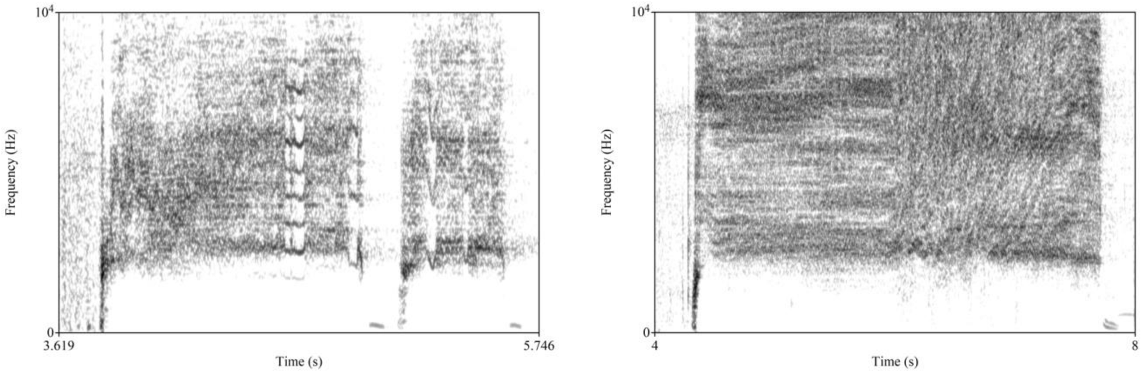

Figure S3. Spectrogram representation of a Scream (SM) vocalization.

Screams are harsh vocalizations characterized by wide-band noise extending up to 15 kHz. They may continue for multiple seconds and contain occasional tonal components with visible overtone structure (most likely to appear at the start or end of the scream). Screams are produced by all age groups both singly and as components of vocal sequences (**Figure S11**).

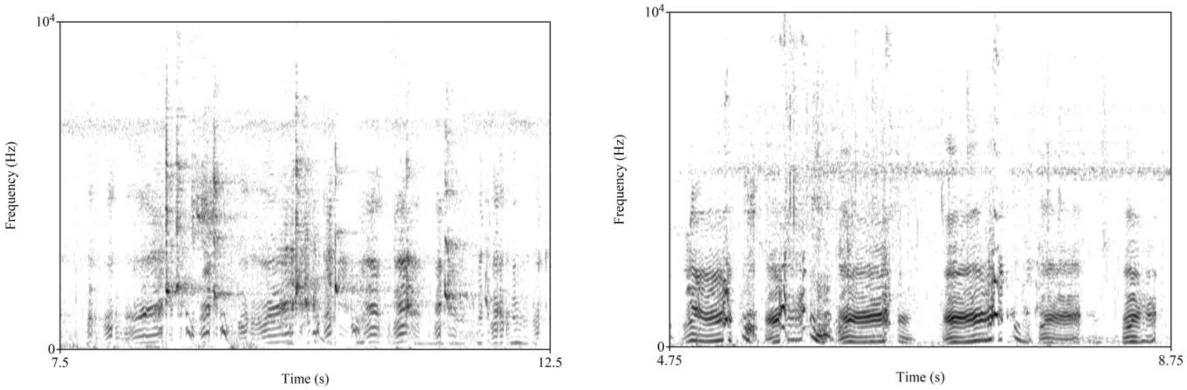

Figure S4. Spectrogram representation of a Growl (GO) vocalization.

Growls are characterized by a low fundamental frequency band with many visible overtones. They are variable in duration and may be made up of a combination of shorter and prolonged elements. They are produced by all age classes and similarly likely to be produced singly or as part of a vocal sequence (**Figure S11**).

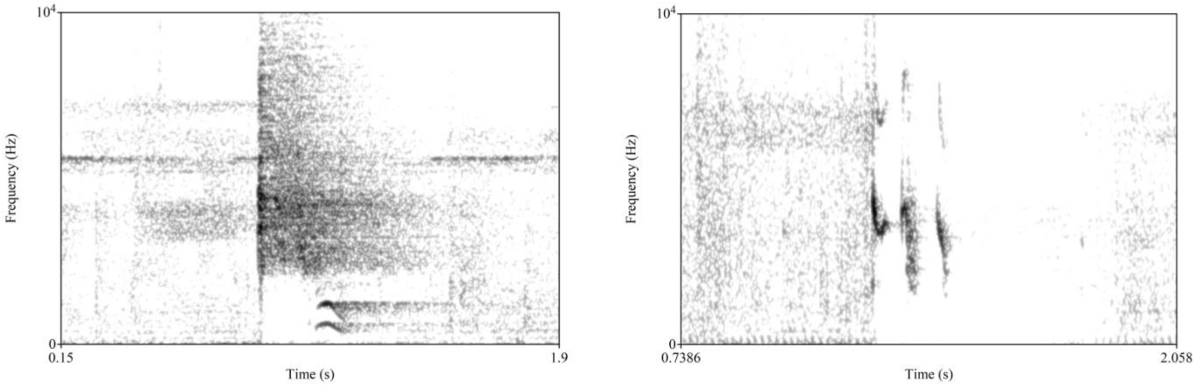

Figure S5. Spectrogram representation of a Shrill Call (SC; Alarm Call) vocalization.

Shrill calls are made up of 1-3 harsh, high-frequency elements. Harmonic structure may be visible, though this is less common. They may or may not contain a final element with a low fundamental frequency and minimal overtone structure. If this final element was included, we still coded it as a single vocal unit, but considered it to be a complex call and excluded it from the model presented in **Figure S22**. Shrill calls were produced by all age groups, albeit at a low rate, and occasionally combined into vocal sequences (**Figure S11**).

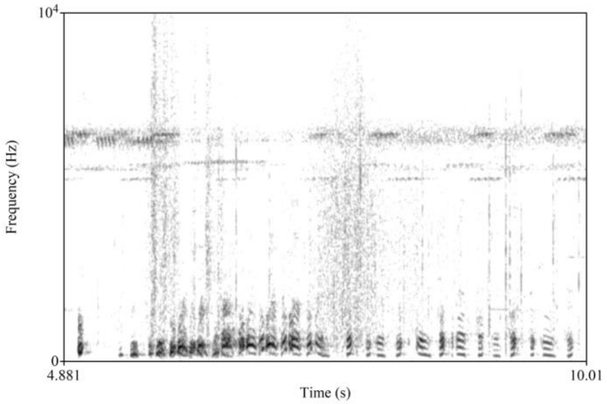

Figure S6. Spectrogram representation of a Vibrato (VI; Copulation Call) vocalization.

The vibrato is a complex call used exclusively by sexually-mature females. It is made up of many variable elements with a low fundamental frequency and rich harmonic structure. The individual elements tend to alternate between shifting their pitch upwards, then downwards (the eponymous "vibrato"). The call may last as long as 10 seconds. This call was also excluded from the model presented in **Figure S22**.

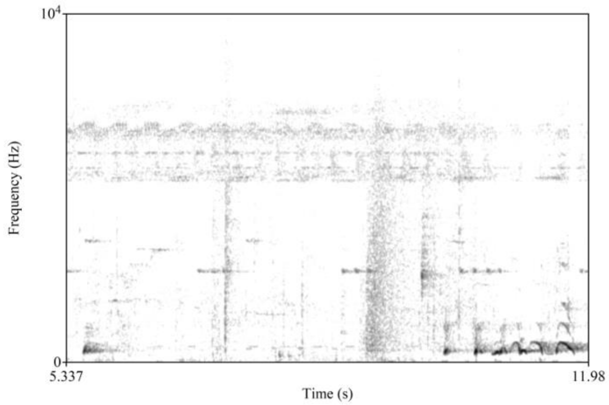

Figure S7. Spectrogram representation of a Long Call (LC; Whoop Gobble) vocalization.

The long call is a loud, low frequency complex call that can be heard from a long distance. They begin with a single low frequency element which is followed by a pause that may extend for several seconds before being concluded by a series of repetitive, low-frequency elements. The pause between the first and final elements violates our usual rule for constituting a vocal unit, but because they are invariably produced in this form we treated them as a special case. Our final sample lacked collected instances of the long call, as it is rarely produced in situations where focal follows are possible. However, our *ad libitum* data collection supports Range and Fischer's claim that they are only produced by adult males (1).

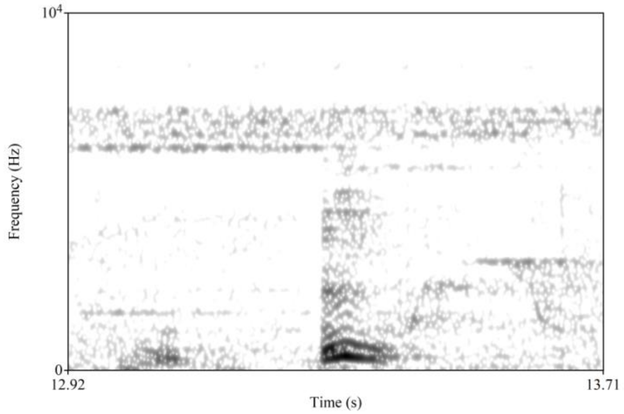

Figure S8. Spectrogram representation of a Hoo (HO) vocalization.

The Hoo consists of a low fundamental frequency with a rich and readily-visible harmonic structure. It is rarely used outside of a vocal sequence and never repeated to form a multi-element unit. The Hoo vocalization was only used by adults in our sample, though our *ad libitum* data contains a single recorded instance of a juvenile producing the call, so it may be present in their repertoire at an earlier developmental point.

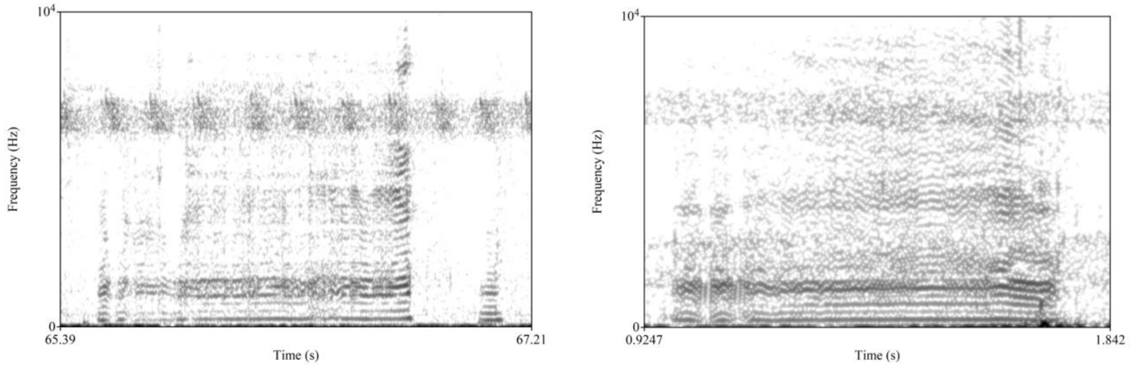

Figure S9. Spectrogram representation of a Whimper (WH) vocalization.

We added one call type not previously described in the literature which we refer to as the whimper. Whimpers are produced exclusively by pre-weaned individuals (less than two years of age), typically in the context of begging interactions with their mothers. They are produced at the highest rate during the weaning period of life, when persistent begging may be required to induce their mother to suckle them. Each unit of a whimper consists of a low fundamental frequency band with many overtones extending to around 2.5 kHz. They may consist of many short individual units blocked together or longer duration units that may be prolonged for multiple seconds.

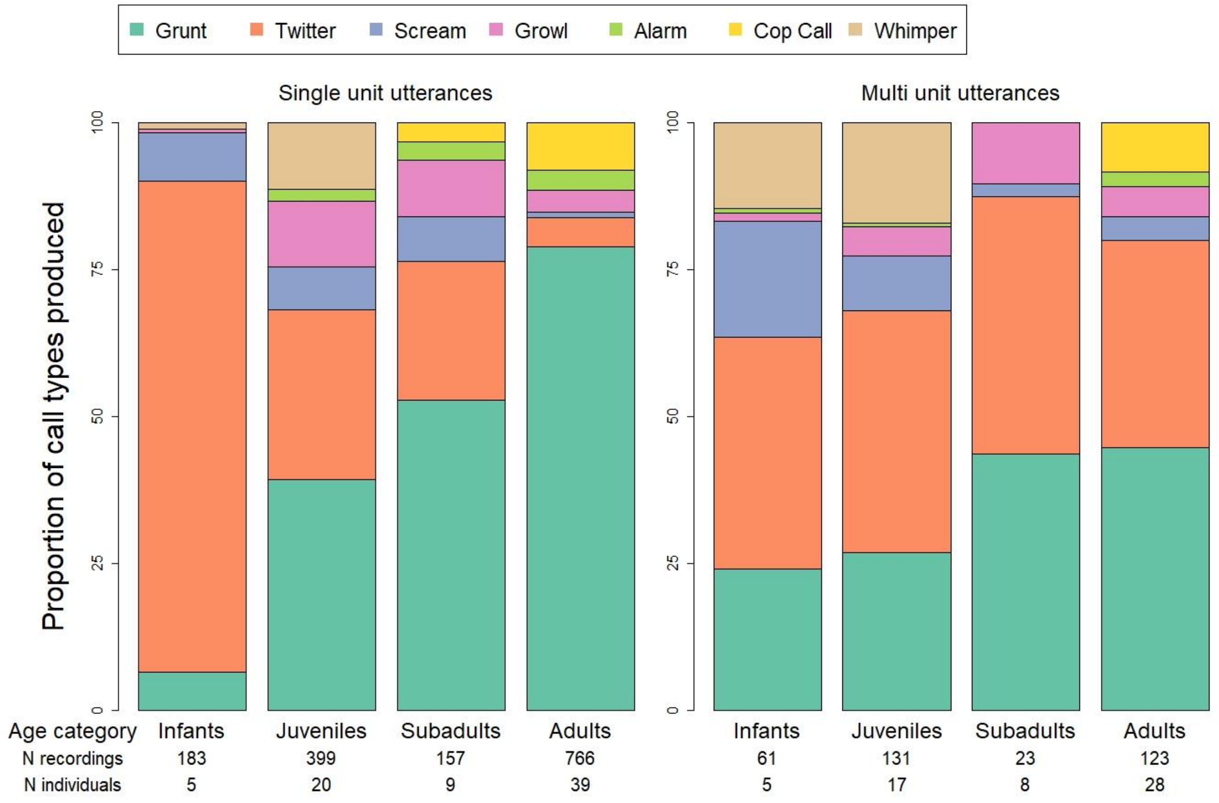

Figure S10. Proportion of vocal unit types produced by each age class

The left-most figure presents the total proportion of vocal units produced by each age class when looking at only those utterances consisting of a single vocal unit. The right-most figure presents the total proportion when looking at only those utterances made up of multiple vocal units (in other words, vocal sequences). Omitted from this figure is the rare vocal unit hoo, of which we only recorded a few examples from our adult age class, and the long call, which our final sample lacked entirely.

A)

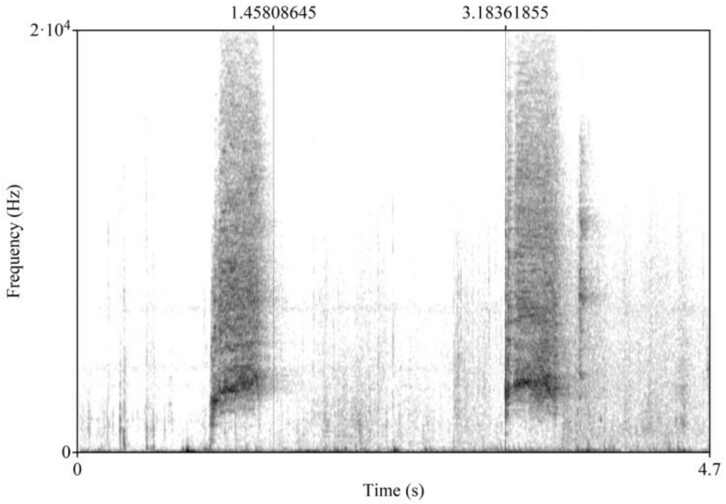

B)

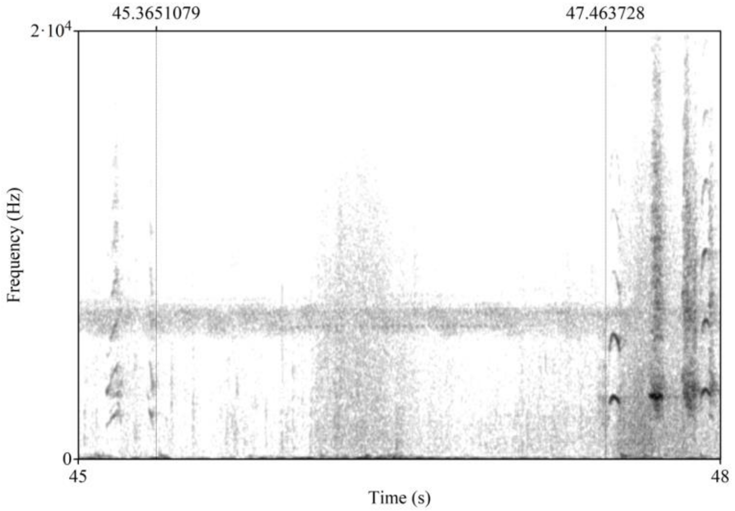

Figure S11. Illustration of vocal unit time cutoff parameters.

The spectrogram in A) consists of two elements of the same type (scream). Because they are 1.73 seconds apart, below the 2-second cutoff for dividing a vocal unit, they are classified as a single vocal unit made up of two elements. Contrast this with the spectrogram in B) where there is a separation of more than 2 seconds between the twitter elements shown, resulting in a classification of two distinct vocal units.

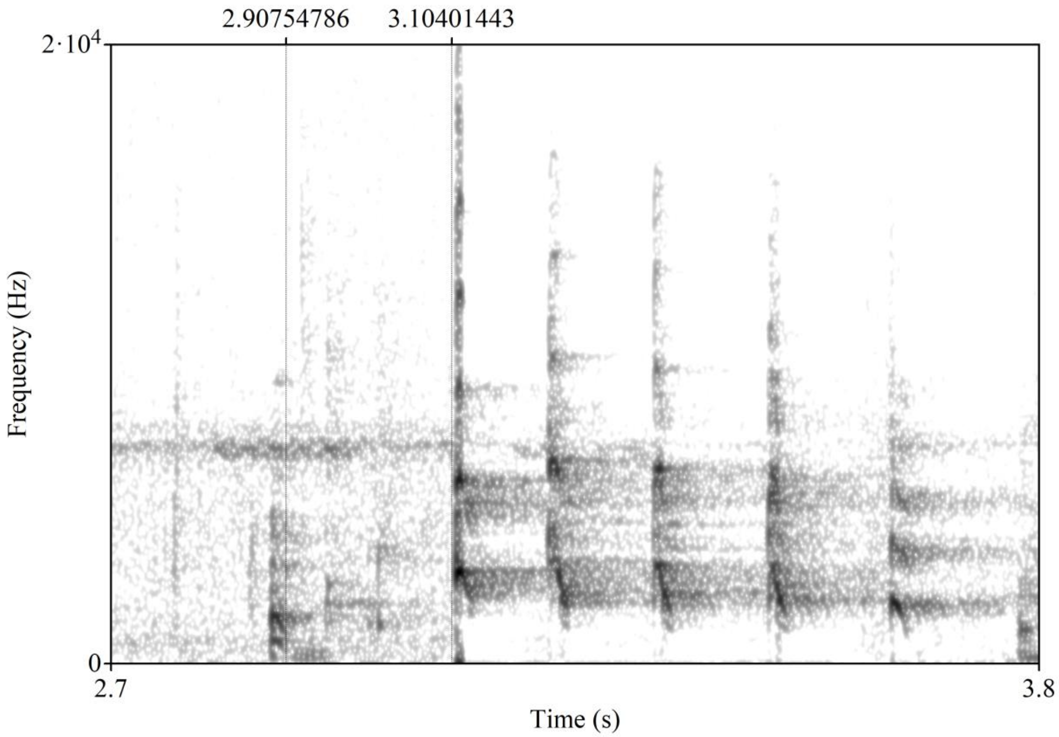

Figure S12. Illustration of vocal sequence cutoff parameters.

Here there are two different vocal units, the grunt and the twitter. Because the time between the two different vocal units is only 0.2 seconds, far below the 1-second cutoff we have defined for sequences, the two vocal units are considered to compose a single vocal sequence (GR-TW).

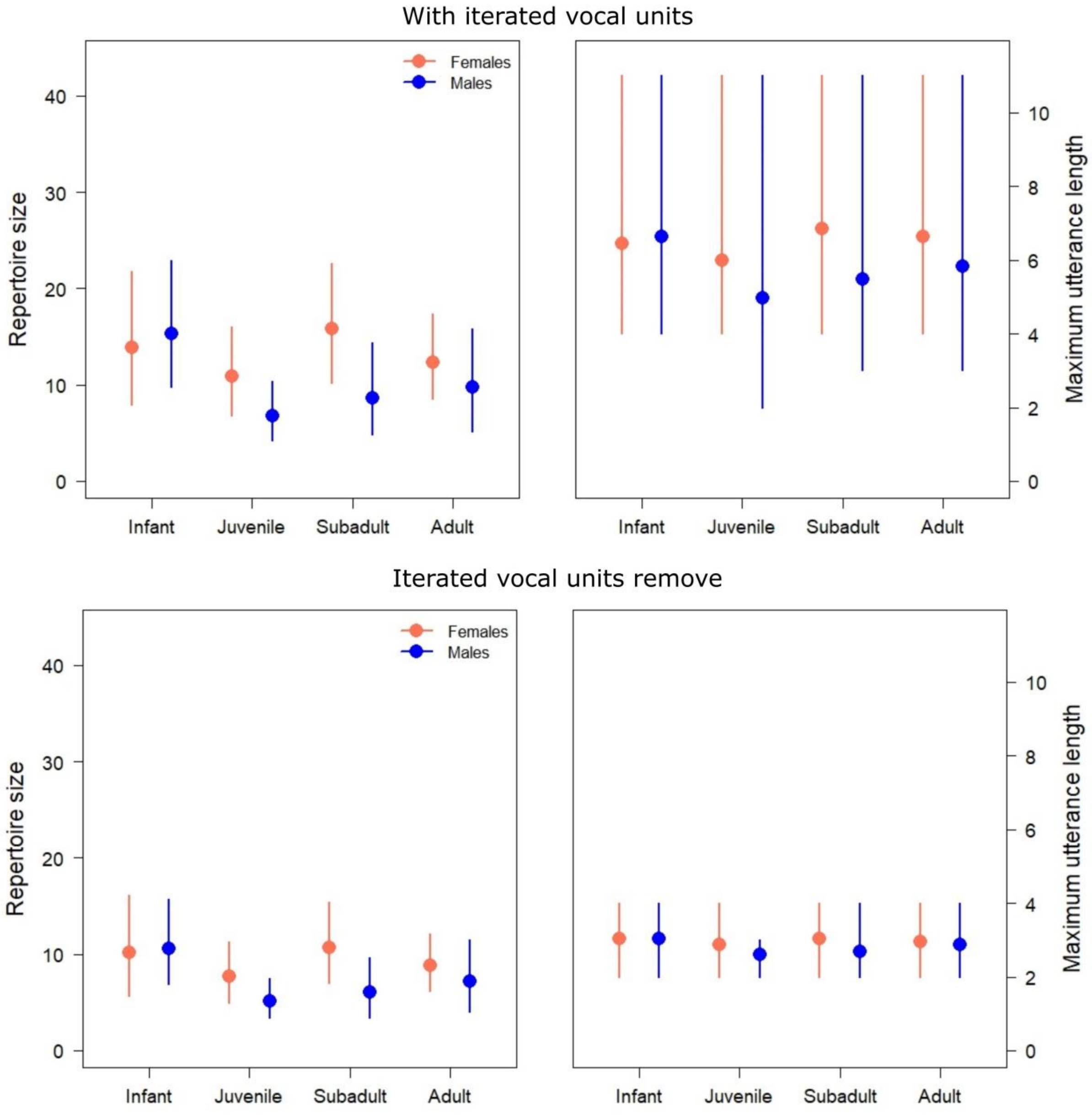

Figure S13. The "whimperless" model, in which single-unit whimpers and sequences containing whimpers were removed from the contributing data set.

These analyses were carried out to determine whether the greater utterance length and diversity observed in infants was attributable to the infant-specific call, whimper. Whimpers are often produced by infants as a component of begging and distress sequences.

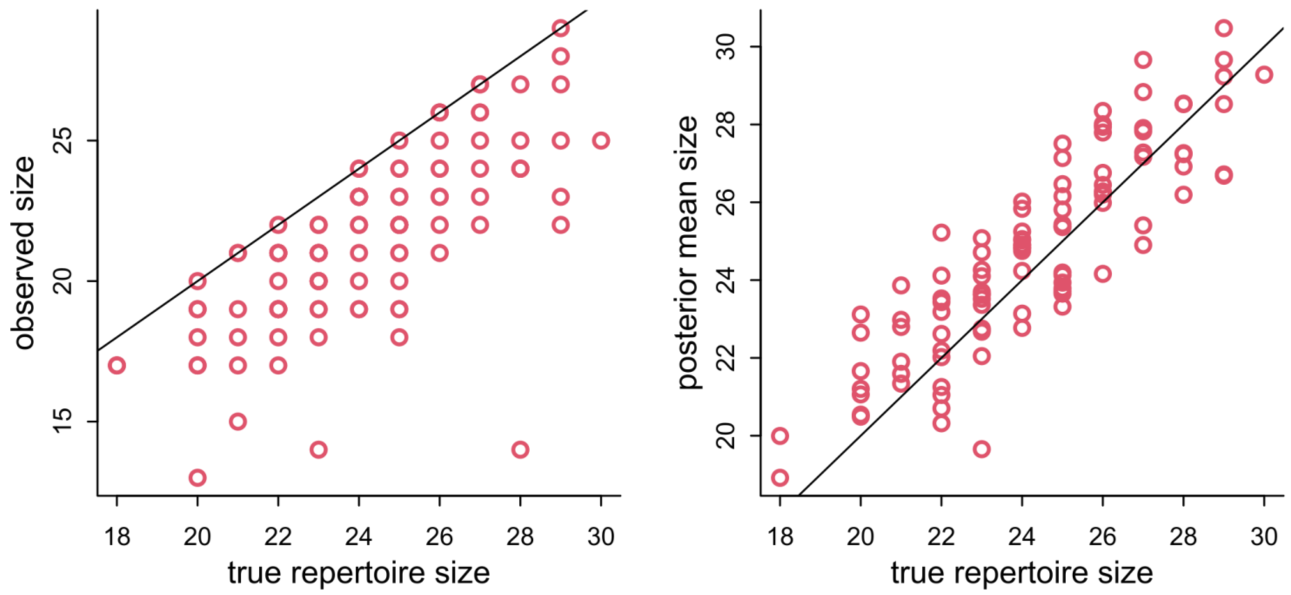

Figure S14. Validation of the statistical model for vocal repertoire size estimation: Observed and model-estimated repertoire size related to simulated ground truth.

This Figure illustrates the result of the simulation run to validate the statistical approach using simulated repertoire size for 50 individuals in a population with 50 possible different vocal utterance types and with an average of 20 recordings per individuals.

The left panel depicts the observed repertoire (from the simulated recordings) versus the ground truth. Each dot represents an individual. Here the observed repertoire size is largely underestimating the ground truth repertoire size.

The right panel depicts the posterior mean estimate of the repertoire size derived from the statistical model versus the ground truth. Here the posterior means are distributed around the 1/1 line indicating a good ability for the model to recover ground truth.

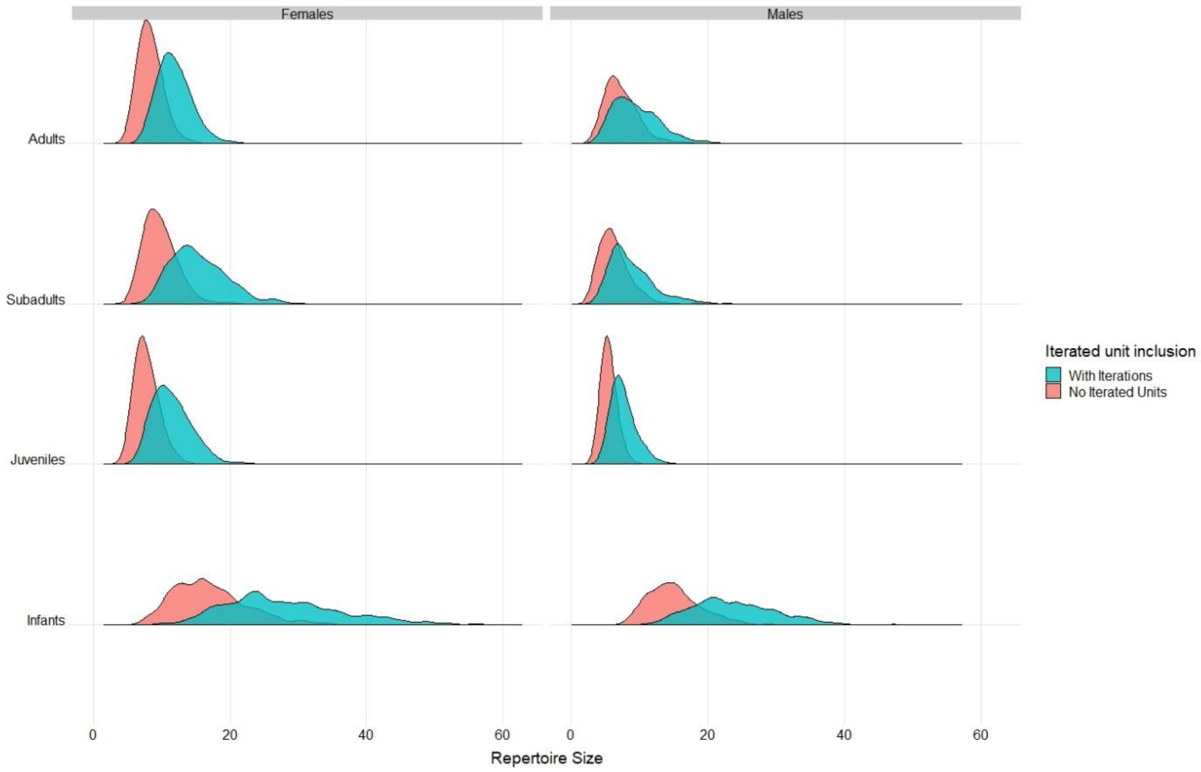

Figure S15. Repertoire size density plot

Density plot of the posterior distribution of the model illustrating the spread of repertoire size predictions for the different age-sex groups, with and without non-adjacent iterated units.

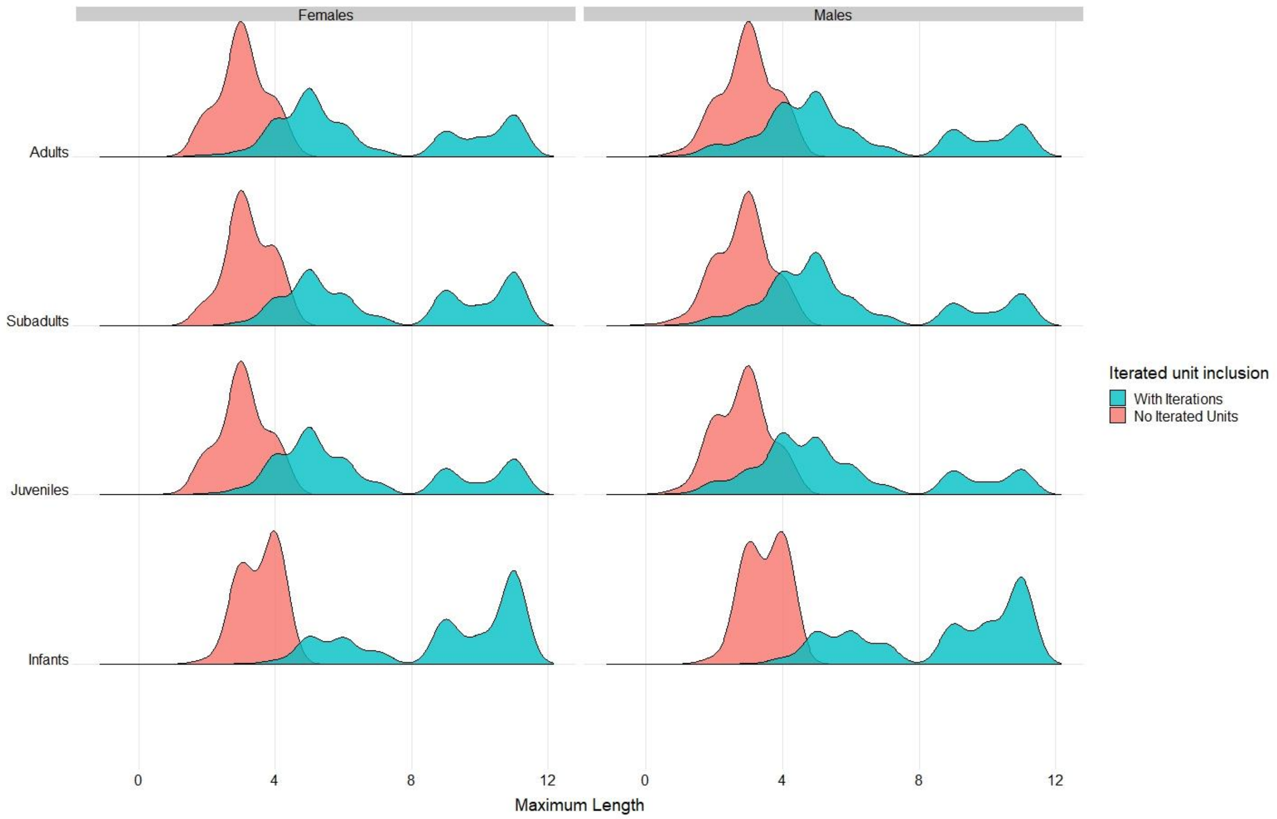

Figure S16. Maximum utterance length density plots.

Density plot of the posterior distribution of the model illustrating the spread of maximum utterance length predictions for the different age groups, with and without iterated units.

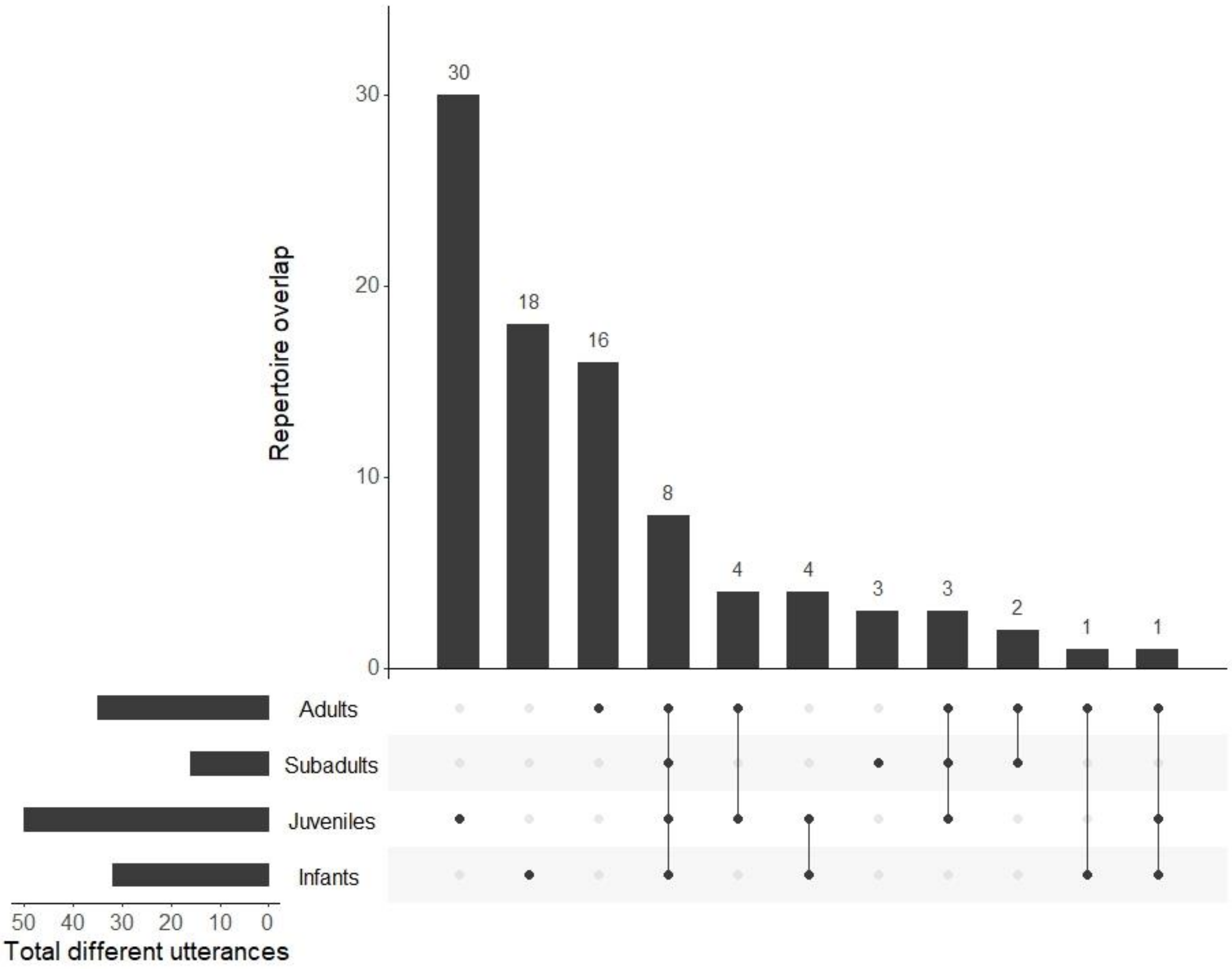

Figure S17. Full repertoire with overlap information with iterated vocal units included.

The above figure is an UpSet Plot showing the number of utterances in the repertoire of each age class that overlap with the repertoire of other age classes. Iterated vocal units have been included in repertoire determination. The main bar plot indicates the number of shared utterances in the vocal repertoire of the age groups indicated by a point below that bar. If there is only a single point, the above bar then represents the utterances unique to that age class. The smaller bar plot in the bottom left-hand corner shows the vocal repertoire size for each age class.

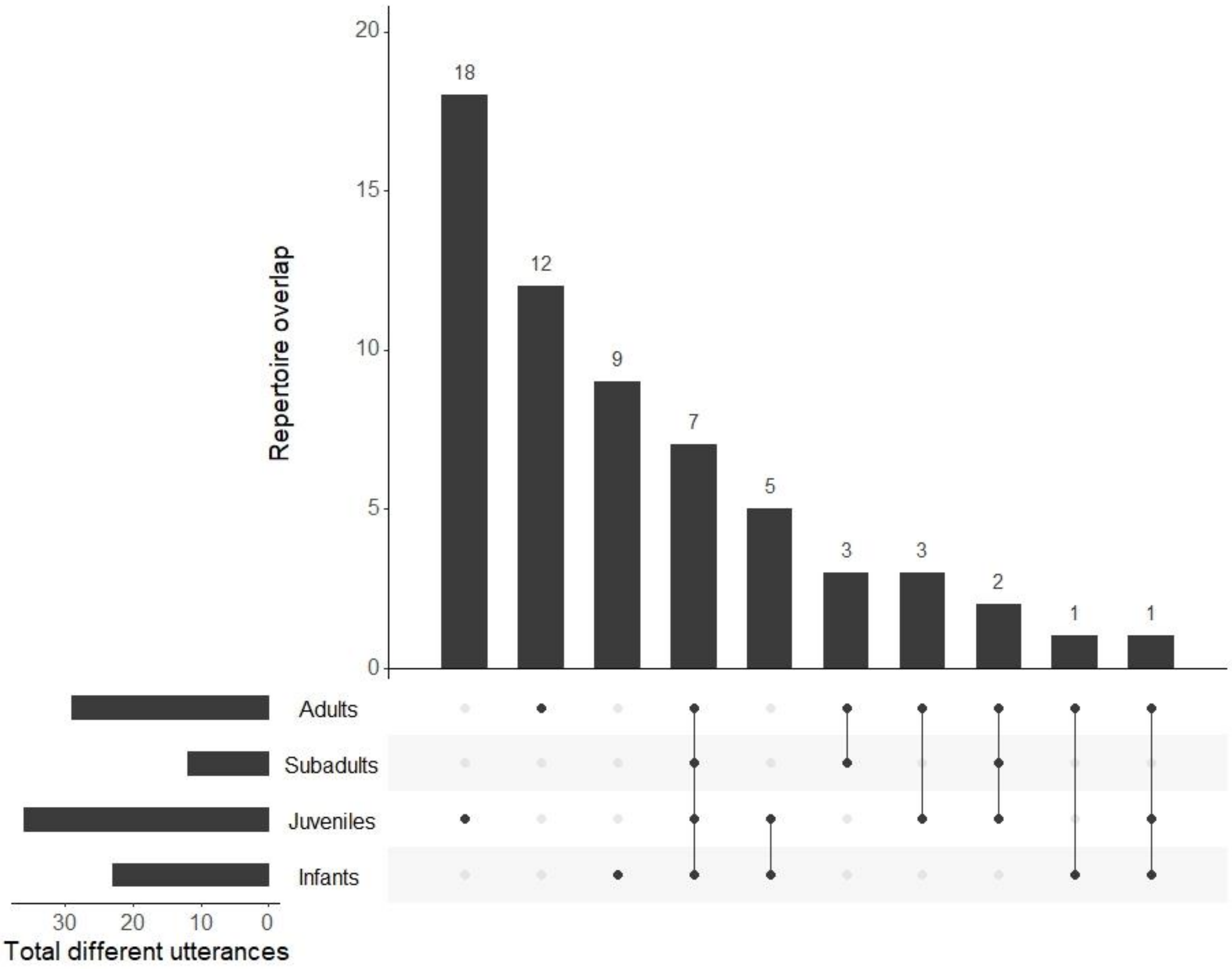

Figure S18. Full repertoire with overlap information with iterated vocal units removed.

The above figure is an UpSet Plot showing the number of utterances in the repertoire of each age class that overlap with the repertoire of other age classes. Iterated vocal units have been excluded in repertoire determination. The main bar plot indicates the number of shared utterances in the vocal repertoire of the age groups indicated by a point below that bar. If there is only a single point, the above bar then represents the utterances unique to that age class. The smaller bar plot in the bottom left-hand corner shows the vocal repertoire size for each age class.

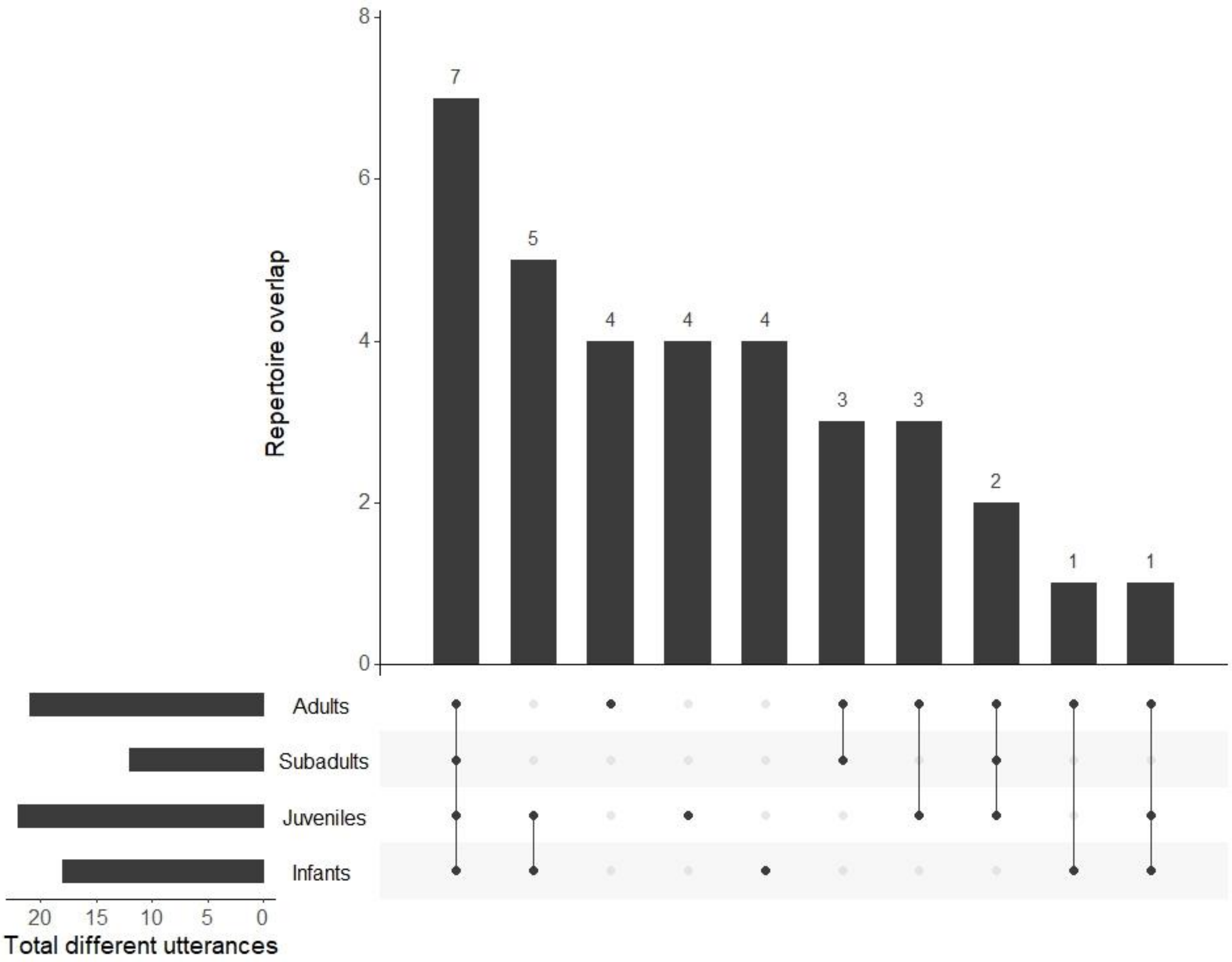

Figure S19. Adult repertoire for utterances with at least two producers with non-adjacent iterated units removed.

Overlap plot: UpSet Plot showing the number of utterances in the repertoire of each age class that overlap with the repertoire of other age classes. Only utterances produced by at least two individuals have been included and iterated vocal units have been excluded in repertoire determination. The main bar plot indicates the number of shared utterances in the vocal repertoire of the age groups indicated by a point below that bar. If there is only a single point, the above bar then represents the utterances unique to that age class. The smaller bar plot in the bottom left-hand corner shows the vocal repertoire size for each age class.

Complete adult vocal repertoire when only utterances produced by at least two individuals have been included and non-adjacent iterated units have been removed:

"GR" "GR_TW" "TW_GR" "GO" "TW" "SC" "SC_GR" "GO_TW" "SM" "VI" "TW_SM" "SM_GO" "VI_GR" "GR_VI" "TW_GO" "GR_GO" "SM_GR" "GR_SC" "GO_GR" "GR_TW_GO" "SC_TW"

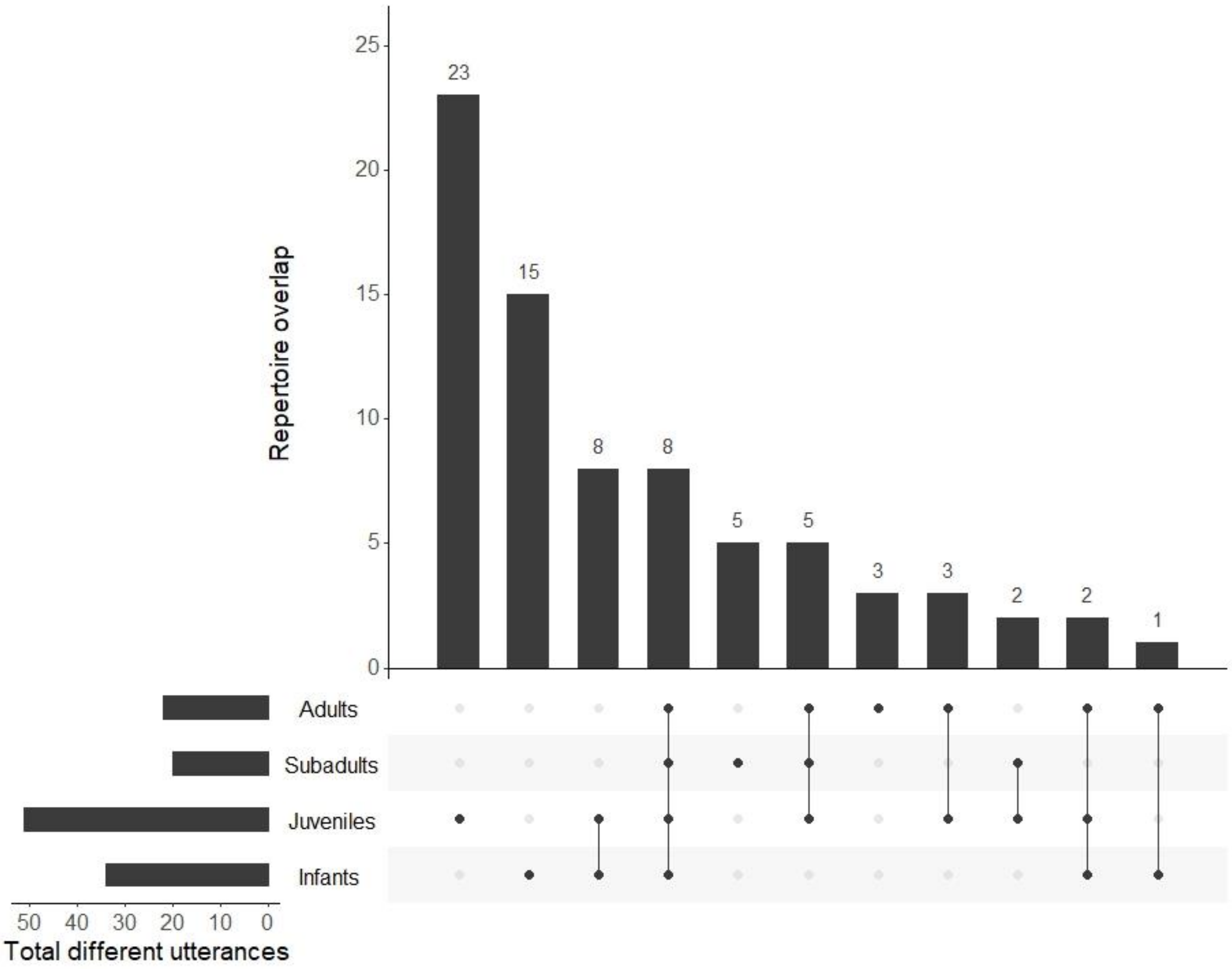

Figure S20. Repertoire overlap for utterances with at least two producers when excluding adult-specific vocal units.

UpSet Plot showing the number of utterances in the repertoire of each age class that overlap with the repertoire of other age classes. Only utterances produced by at least two individuals have been included and all utterances including vocal units unique to adults have been excluded from the contributing sample. Iterated vocal units have been included in repertoire determination. The main bar plot indicates the number of shared utterances in the vocal repertoire of the age groups indicated by a point below that bar. If there is only a single point, the above bar then represents the utterances unique to that age class. The smaller bar plot in the bottom left-hand corner shows the vocal repertoire size for each age class.

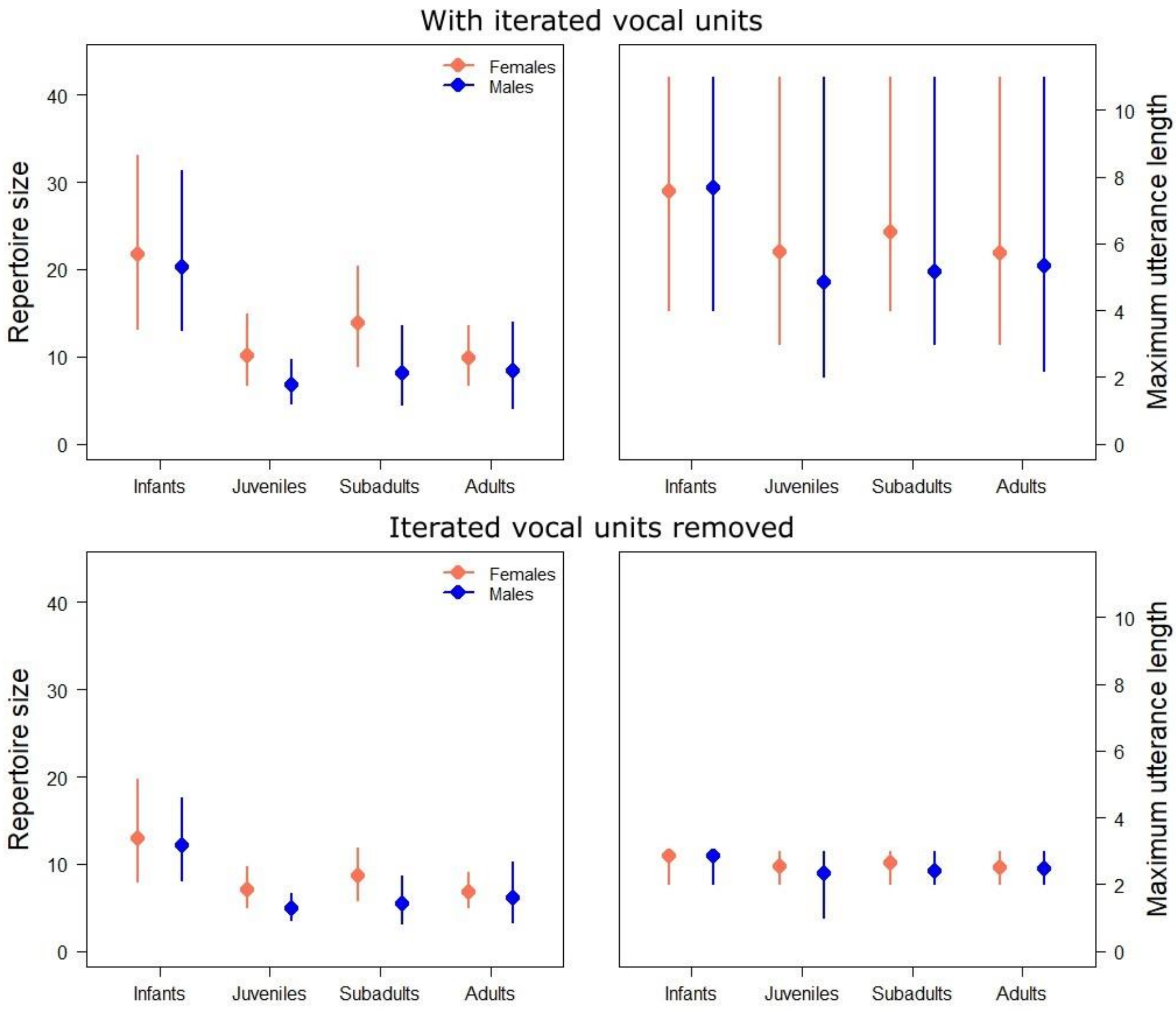

Figure S21. The conservative case model, in which we excluded all utterances that were not produced by at least two individuals. This resulted in the removal of 23 utterances, reducing the total sample from 1844 to 1821 and the total observed utterances from 90 to 67.

The plot depicts model estimates for mean repertoire size and maximum utterance length broken down by age class and sex for the conservative model. Points represent mean estimates while the extending lines express the confidence interval. The top two plots include instances of iterated call units within an utterance while the bottom two plots remove such instances. A corresponding decrease in the estimated repertoire size when compared to our main results is apparent in the plots presented here, but the general pattern observed in our main paper remains intact.

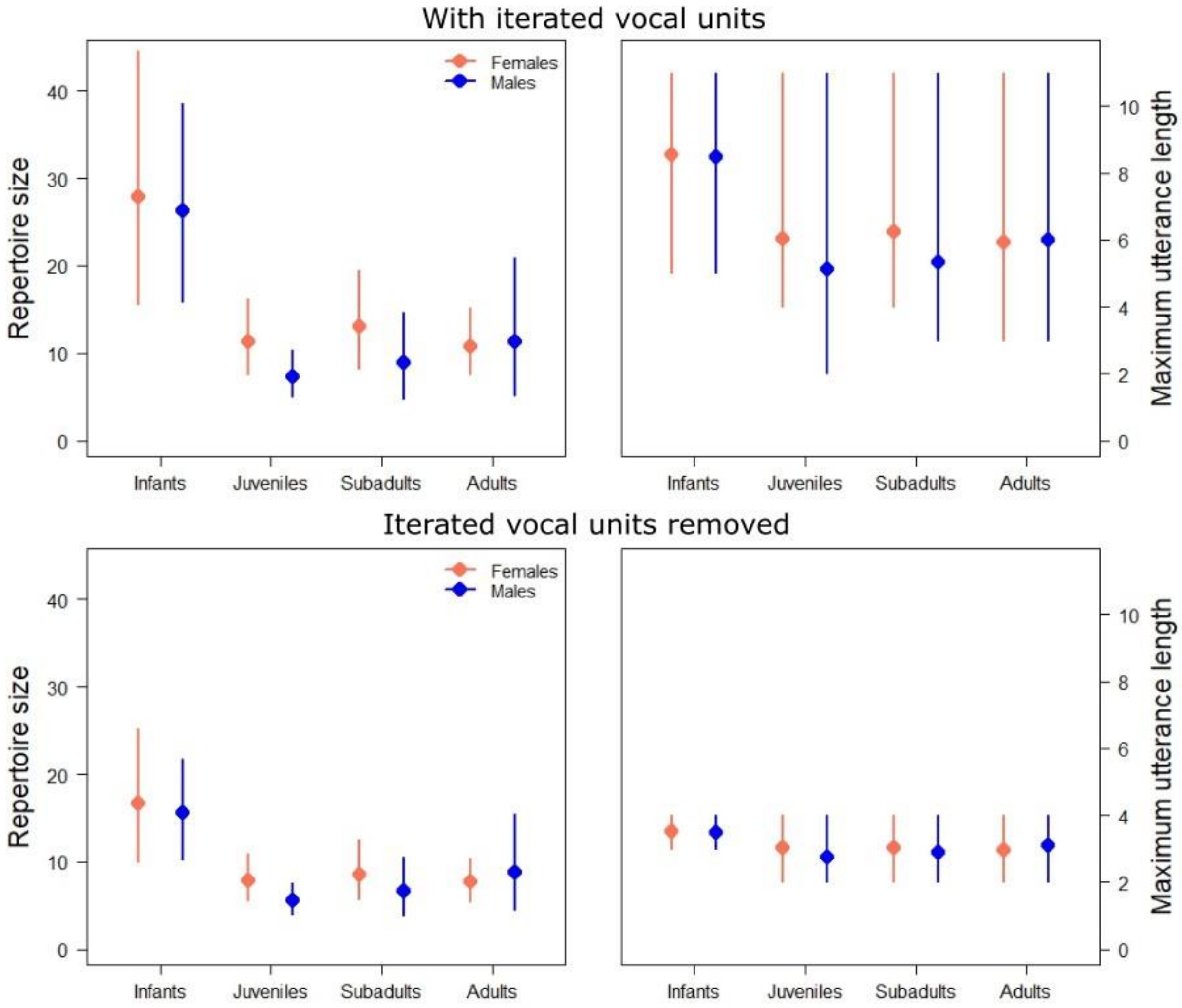

Figure S22. The complex call exclusion model, in which all call types made up of diverse elements have been removed from the contributing sample.

Some of the calls in the mangabey repertoire (namely, vibrato, long calls, and shrill calls) are composed of what are debatably multiple unit types. They are classed as a single call on the basis that these units do not occur independently, only in the context of the other units composing this specific call. Some studies in the primate literature do not acknowledge this distinction, so to aid in comparison with such studies we present here the plotted output of the model when it is run with a data set excluding these calls.

The plot depicts model estimates for mean repertoire size and maximum utterance length broken down by age class and sex from a model excluding complex calls. Points represent mean estimates while the extending lines express the confidence interval. The top two plots include instances of iterated call units within an utterance while the bottom two plots remove such instances. The pattern observed in the full data set remains essentially unchanged in this reduced data set.

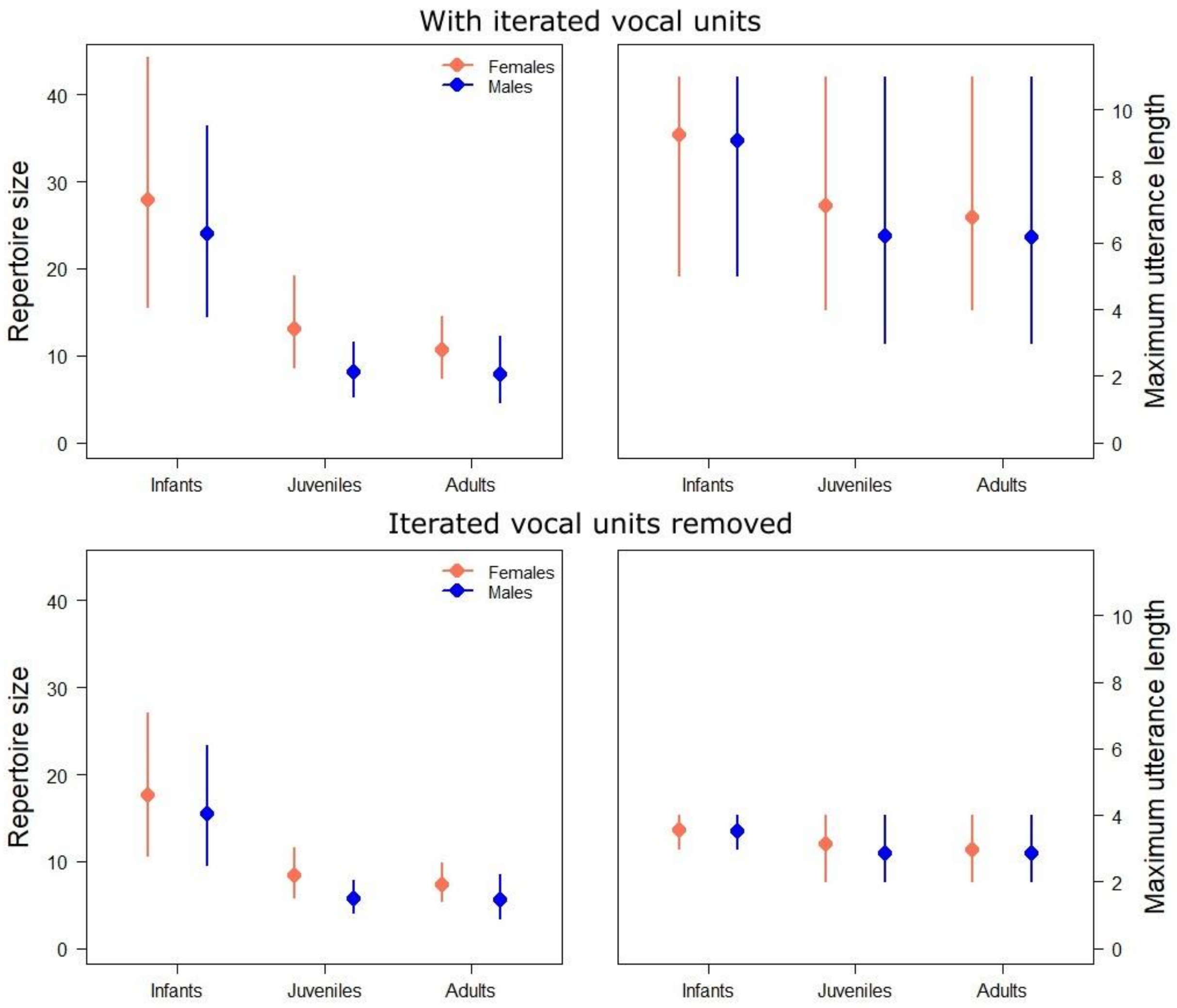

Figure S23. The “no subadult class” model, in which individuals that were classed as subadults in our main results were reclassified as adults.

We made the decision in our main paper to divide the mangabey lifespan into four distinct age class, but much of the existing literature does not make use of the subadult age class. To aid in comparison to such studies as well as demonstrate that the pattern we observed is still present even in when this class is not included, we present here plots of the output from the model when it is run without the subadult class. Individuals previously classified as subadult were reclassified as adults to create the data set for this model.

The plot depicts model estimates for mean repertoire size and maximum utterance length broken down by age class and sex. Points represent mean estimates while the extending lines express the confidence interval. The top two plots include instances of iterated call units within an utterance while the bottom two plots remove such instances. The pattern we observed in our main results remains essentially unchanged in this data set.

References

1. Range F, Fischer J. Vocal Repertoire of Sooty Mangabeys (Cercocebus torquatus atys) in the Taï National Park. Ethology. 2004;110(4):301–21.

2. Range F, Noë R. Familiarity and dominance relations among female sooty mangabeys in the Taï National Park: Relations Among Female Sooty Mangabeys. Am J Primatol. 2002 Mar;56(3):137–53.

3. Ehardt CL. Absence of strongly kin-preferential behavior by adult female sooty mangabeys (*Cercocebus atys*). Am J Phys Anthropol. 1988 Jun;76(2):233–43.

4. Range F. Social behavior of free-ranging juvenile sooty mangabeys (Cercocebus torquatus atys). Behav Ecol Sociobiol. 2006;59(4):511–20.

5. Gust DA, Gordon TP. Male age and reproductive behaviour in sooty mangabeys, Cercocebus torquatus atys. Anim Behav. 1991 Feb;41(2):277–83.

6. Gust DA, Busse CD, Gordon TP. Reproductive parameters in the sooty mangabey (*Cercocebus torquatus atys*). Am J Primatol. 1990 Jan;22(4):241–50.

7. Hadidian J, Bernstein IS. Female reproductive cycles and birth data from an Old World Monkey colony. Primates. 1979 Jul;20(3):429–42.

8. Mann R, Daniel V, Gould G, Antonio S. Developmental patterns of serum luteinizing hormone, gonadal and adrenal steroids in the sooty mangabey (Cercocebus atys). 1983;284:279–84.
