# Supplementary material for: Vocal sequence diversity and length remain stable across ontogeny in a catarrhine monkey (Cercocebus atys)
Source: Commun Biol. 2025 Mar 20;8:465. doi: 10.1038/s42003-025-07922-2 (PMC11926236; doi:10.1038/s42003-025-07922-2)
Supplement: Supplementary file 5 — Description of Additional Supplementary Files [file 42003_2025_7922_MOESM5_ESM.pdf]

Description of Additional Supplementary Files

File name: Supplementary Data 1

Description: Utterance frequency table showing how many times each particular utterance in our data set occurred.

File name: Supplementary Data 2

Description: Mean probability estimates and estimated call rate for each utterance in the mangabey repertoire.
